# Microbial communities in the rhizosphere of three *Mentha* species: Links to soil properties and essential oil profiles

**DOI:** 10.1371/journal.pone.0354132

**Published:** 2026-07-31

**Authors:** Yasmine Wazzani, Szilvia Tavaszi-Sárosi, Katalin Patonay, Ferenc Olasz, Ákos Juhász, Katalin Posta

**Affiliations:** 1 Agribiotechnology and Precision Breeding for Food Security National Laboratory, Institute of Genetics and Biotechnology, Hungarian University of Agriculture and Life Sciences, Gödöllő, Hungary; 2 Doctoral School of Natural Sciences, Hungarian University of Agriculture and Life Sciences, Gödöllő, Hungary; 3 Department of Medicinal and Aromatic Plants, Institute of Horticultural Science, Hungarian University of Agriculture and Life Sciences, Budapest, Hungary; 4 Food and Wine Knowledge Centre, Eszterházy Károly Catholic University, Eger, Hungary; University of Salento: Universita del Salento, ITALY

## Abstract

*Mentha* species are widely cultivated aromatic plants valued for their essential oils and antimicrobial properties. However, despite their agricultural and pharmacological significance, limited information is available on how different *Mentha* species influence rhizosphere microbial communities and their relationships with soil physicochemical parameters and essential oil composition. In this study, we examined the rhizosphere microbiota of three closely related taxa – *Mentha* × *villosa* B10, *M. spicata* B17, and *M. suaveolens* J17 – cultivated under uniform field conditions. Rhizosphere and bulk soils were analyzed for physicochemical properties, microbial composition (16S rRNA, ITS sequencing), essential oils (gas chromatography-mass spectrometry), and arbuscular mycorrhizal colonization. Bacterial communities were dominated by the phyla Actinomycetota, Pseudomonadota, Acidobacteriota, Bacillota, and Chloroflexota, while fungal communities were primarily composed of Ascomycota, Mortierellomycota, Basidiomycota, and Rozellomycota. Rhizosphere soils exhibited higher fungal diversity than bulk soils, with Glomeromycota detected exclusively in rhizosphere. Microbial community composition differed significantly among *Mentha* taxa: *M. spicata* B17 displayed the lowest bacterial diversity, the most distinct microbial assemblages, and the highest arbuscular mycorrhiza colonization. Soil properties – particularly humus content, phosphorus, potassium, and sodium – were strongly correlated with bacterial diversity, while fungal communities showed weaker associations. Integration of essential oil data revealed genotype-dependent chemical profiles: *Mentha* × *villosa* B10 and *M. spicata* B17 were characterized by high proportions of L-carvone and limonene, whereas *M. suaveolens* J17 was dominated by cis-piperitone epoxide and piperitenone oxide. Together, these findings demonstrate that even closely related *Mentha* cultivars can harbor distinct rhizosphere microbiota, associated with both plant chemical traits and soil characteristics. This study highlights the complex interactions between aromatic plants, soil chemistry, and microbial communities, offering novel insights into plant-soil-microbe interactions in medicinal and aromatic crop systems.

## Introduction

The rhizosphere, the narrow zone of soil influenced by plant roots, represents a dynamic interface where complex biological, chemical, and physical interactions shape plant-microbe relationships [1,2]. This zone is nutrient-rich due to the accumulation of various plant exudates, such as amino acids and sugars, which serve as abundant energy and nutrient sources for microbes [3]. This microenvironment hosts diverse microbial communities including bacteria, fungi, archaea, and protists, many of which play essential roles in nutrient cycling, plant growth promotion, and protection against pathogens [4,5]. The composition and function of rhizosphere microbiota are influenced by numerous factors, including plant genotype, developmental stage, soil type, and agricultural management practices [6–8]. Among the microbial constituents, arbuscular mycorrhizal fungi, belonging predominantly to the phylum Glomeromycota, form symbiotic associations with roots of most terrestrial plants, enhancing water and nutrient uptake, particularly phosphorus, while receiving carbon in return [9,10]. The composition of microbial communities in the rhizosphere is not static, but responds dynamically to plant-specific traits, developmental stages, and root exudate profiles [11]. Recent advances in metagenomics have revolutionized our understanding of rhizosphere microbiota. Unlike traditional culture-based methods, sequencing allows for high-resolution, culture-independent analysis of both taxonomic diversity and functional potential. This approach enables researchers to detect both abundant and rare microbial taxa, including those unculturable by conventional means [12,13].

Members of the *Lamiaceae* family, particularly aromatic and medicinal species such as *Mentha*, *Thymus*, and *Salvia*, are well known for their production of bioactive secondary metabolites, including essential oils (EOs) [14]. The biologically active compounds of plant origin can enter the soil not only through root exudates but also via fallen leaves, stem residues, and gaseous emissions [15]. These metabolites can either stimulate or inhibit microbial growth, and in the case of essential oil-producing plants, contribute to the formation of phytogenic fields with distinct rhizosphere microbial communities compared to bulk soil [16]. Long-term cultivation of medicinal and aromatic plants can significantly impact soil microbial communities, raising concerns about soil health and productivity; as a result, the structure and function of the soil microbiome may gradually shift, highlighting the need for further research on how long-term aromatic plant cultivation influences soil microbial diversity [17].

*Mentha* species are rich in EOs and have attracted significant interest due to their strong antioxidant activity, low toxicity, and broad range of health benefits. They exhibit potent antimicrobial effects against pathogens [18–21]. Despite the pharmacological and industrial relevance of *Mentha* species and the recognized roles of root exudates in shaping soil microbiota, surprisingly few studies have addressed the composition and structure of rhizosphere microbial communities in these plants. Most previous investigations have focused on the antimicrobial activity of EOs *in vitro*, rather than their ecological effects *in situ* [22]. A few experimental studies have investigated how the addition of leaf residues or EO extracts to soil influences microbial activity, revealing both stimulatory and suppressive effects depending on the context [23]. Similarly, some *Lamiaceae* species, including *Thymus* and *Lavandula*, have been examined for their impact on soil microbial dynamics, with findings suggesting a selective enrichment of microbial taxa potentially adapted to EO-rich environments [24,25]. However, comprehensive community-level studies of the native rhizosphere microbiota of *Mentha* (and *Lamiaceae*) species using high-throughput sequencing are still scarce. It remains unclear whether closely related *Mentha* taxa grown under similar field conditions harbor distinct rhizosphere microbial communities and how such patterns are associated with soil physicochemical properties and cultivar-specific chemotypes.

A few recent publications have begun to fill this gap. For example, Zharkova et al. [26] examined bacterial community structures in the rhizosphere of various *Lamiaceae* species, including mint, and found that plant genotype significantly influenced microbial composition. Checcucci et al. [24] further demonstrated that essential oil-producing *Thymus* species exhibit distinct bacterial communities in their rhizospheres, possibly due to the antimicrobial pressure exerted by plant volatiles. Deng et al. [25] and Li et al. [11] investigated fungal and bacterial communities, respectively, in lavender and *Salvia* rhizospheres, highlighting shifts in community composition over time and across phenological stages. However, these studies have primarily focused on individual species or time-course dynamics, with limited comparative insights across multiple *Mentha* taxa.

In parallel, arbuscular mycorrhizal associations in *Mentha* species have also gained attention, as studies have shown that arbuscular mycorrhizal fungi (AMF) inoculation can enhance plant nutrition and increase EO yield and compound concentration, particularly under nutrient-limited conditions [27–30]. Mycorrhizal colonization may also influence root architecture, soil pH, and enzymatic activity in the rhizosphere. Furthermore, interactions between AMF and host plant can affect the structure and functional potential of the rhizosphere microbial community [27].

To date, no comprehensive study has simultaneously explored the rhizosphere microbial community structure and mycorrhizal colonization across different *Mentha* species. Understanding such interactions is critical not only for unraveling plant-microbe coevolution but also for optimizing cultivation strategies for medicinal and aromatic plants.

The aim of this study was to examine whether closely related *Mentha* taxa grown under similar field conditions harbor distinct rhizosphere bacterial and fungal communities. Specifically, we aimed to (i) compare soil physicochemical properties and aerial-tissue EO profiles among three *Mentha* species (*M. × villosa*, *M. spicata*, and *M. suaveolens*), (ii) characterize bacterial and fungal community composition, diversity, and differentially abundant taxa in the rhizospheres of these species and in adjacent bulk soil, and (iii) identify soil physicochemical variables and major EO constituents statistically associated with the observed microbial community patterns.

## Materials and methods

### Ethics statement

This study did not involve human participants or animals and therefore did not require approval from an Institutional Review Board or Ethics Committee. Plant and rhizosphere soil samples were collected from the experimental field of the Department of Medicinal and Aromatic Plants, Hungarian University of Agriculture and Life Sciences, on institutional land owned and managed by the university, and no specific permits were required for sampling. No endangered or protected species were involved. Informed consent was not applicable to this study.

### Study area and soil sampling

Plant and rhizosphere soil samples were collected from the experimental field of the Department of Medicinal and Aromatic Plants, Hungarian University of Agriculture and Life Sciences, in late July 2024, during the full flowering phenological stage of the plants. The standing cultures were established by vegetative propagation of mother plants in 2022. Three *Mentha* cultivars were selected for the study: *M.* × *villosa* B10, *M. spicata* B17, and *M. suaveolens* J17, with accession codes referring to entries in our institutional gene bank. Each cultivar was grown in 1.5 × 2.5 m plots, separated by 1.5 m-wide inter-row strips. These strips are regularly maintained by mechanical weeding or shallow tillage and thus remain free of both cultivated and spontaneous vegetation.

Rhizosphere soil samples were collected from a depth of 10–15 cm using sterile tools, in three replicates per cultivar. Bulk soil samples were collected as controls from the vegetation-free inter-row strips separating the *Mentha* plots, located approximately 50 cm from the nearest plant rows. As these strips are maintained by shallow mechanical cultivation during the growing season, they represent managed, disturbed inter-row soil rather than undisturbed natural bulk soil. Samples were placed into sterile 50 mL Falcon tubes and kept on ice during transport to the laboratory. Upon arrival, 0.1 g of soil was aliquoted into sterile 1.5 mL Eppendorf tubes and stored at –80 °C until DNA extraction.

### Soil physical and chemical properties determination

The physicochemical properties of rhizosphere and bulk soils were determined according to Hungarian national standards (MSZ), and the applied MSZ standards, together with their corresponding or comparable international standards, are summarized in S1 Table. The physical classification of the soils was based on the Arany-type plasticity index (KA), determined according to the Hungarian standard MSZ 08–0205:1978. Soil pH was measured in 1 M KCl suspension following the MSZ-08–0206:1978. The humus content was assessed using potassium dichromate oxidation, based on MSZ 21470–52:1983. Available phosphorus (P_2_O_5_), sodium (Na⁺) and potassium (K₂O) contents were extracted using ammonium-lactate solution (AL method) and determined according to MSZ 20135:1999. The nitrate and nitrite nitrogen content (NO_3_⁻ + NO_2_⁻) was extracted with 1 M KCl and analyzed according to MSZ 20135:1999. The AL-extractable P_2_O_5_, K_2_O and Na⁺ values represent plant-available or exchangeable forms commonly used in agronomic assessments.

### Essential oil extraction and GC–MS analysis

For essential oil extraction, approximately 200 g of flowering stems (ca. 40 cm long) were harvested and shade-dried for three weeks. The dried leaves were separated from the stems and were subjected to hydro-distillation (10 g per replicate, three replicates) with 500 mL of distilled water for 2.5 hours using a Clevenger-type apparatus, following the 7th edition of the Hungarian Pharmacopoeia. Following distillation, residual water in the essential oil was removed using anhydrous sodium sulfate. The obtained essential oils were stored in airtight vials at 4 °C until further analysis.

Gas chromatography–mass spectrometry (GC–MS) analysis was performed on each EO sample using an Agilent Technologies 6890N GC system coupled with a 5975 inert mass selective detector and equipped with an HP-5MS capillary column (30 m × 0.25 mm i.d., 0.25 μm film thickness). The oven temperature was programmed to start at 60 °C, then increased at a rate of 3 °C/min up to 240 °C, and held at the final temperature for 5 minutes. Helium was used as the carrier gas at a constant flow rate of 1 mL/min. The injector and detector temperatures were both set at 250 °C, with a split injection ratio of 30:1. Essential oil samples were diluted in n-hexane (10 μL EO in 1 mL solvent), and 0.2 μL of this solution was injected for analysis.

Compound identification was based on linear retention indices (LRI) calculated relative to a homologous series of n-alkanes (C9–C23) using the Van den Dool and Kratz (1963) equation [31], comparison with literature retention index data, and mass spectral matching against commercial libraries (NIST and Wiley). No novel compounds were detected, and the identified constituents were consistent with earlier reports on *Mentha* EO composition.

It should be noted that EOs were extracted from aerial plant tissues and were not directly measured in the rhizosphere soil or as root exudates. Therefore, associations between essential oil composition and microbial communities should be interpreted as correlations with plant chemotype rather than direct measurements of rhizosphere chemical drivers.

### Arbuscular mycorrhizal colonization determination

In parallel with the rhizosphere soil samples, root samples were also collected to assess arbuscular mycorrhizal (AM) colonization. The staining procedure followed the ink–vinegar method originally described by Vierheilig *et al*. (1998) [32], with minor modifications. Approximately 500 mg of fine roots per sample (in triplicate) were gently washed under running tap water to remove adhering soil particles, then cut into segments of approximately 1 cm in length. Root segments were cleared in 10% (w/v) potassium hydroxide (KOH) at 90 °C for 30 minutes and subsequently rinsed with distilled water. Samples were then acidified in 5% (v/v) acetic acid for 5 minutes. Staining was performed in a 5% (v/v) solution of commercially available black ink (Pelikan®) dissolved in 5% acetic acid, by heating the samples at 90 °C for 15–20 minutes. Stained roots were stored in acidified glycerol (glycerol:acetic acid:water, 1:1:1, v/v/v) until microscopic analysis.

For the assessment of mycorrhizal colonization, stained root fragments were mounted on slides and examined under a compound light microscope at 200 × magnification. The gridline intersect method, as described by Giovannetti and Mosse [33], was used to quantify colonization by recording the presence or absence of AM fungal structures (hyphae, arbuscules, vesicles) at regular gridline intersections. At least 100 intersects were analyzed per sample to ensure statistical reliability.

### DNA extraction and amplicon sequencing of rhizosphere soil samples

Genomic DNA was extracted from 100 mg of rhizosphere soil (three replicates per cultivar) using the ZymoBIOMICS 96 MagBead DNA Kit (Zymo Research, Irvine, CA, USA), following the manufacturer’s instructions. DNA concentration was measured with the Quant-iT dsDNA BR Assay Kit (Thermo Fisher Scientific, Waltham, MA, USA) using an Infinite Pro 200 F Nano+ Fluorescent Plate Reader (Tecan, Männedorf, Switzerland).

Bacterial and fungal community composition was assessed via high-throughput sequencing of the 16S rRNA and internal transcribed spacer (ITS) gene regions, respectively, performed on an Illumina NextSeq 2000 platform at Xenovea Ltd. (Szeged, Hungary). The V3–V4 region of the bacterial 16S rRNA gene was amplified using tagged primers, as described by Klindworth et al. (2013) [34]. Fungal ITS regions were amplified using tagged ITS1F and ITS4R primers, according to protocols provided in the Illumina 16S and Fungal Metagenomic Sequencing Library Preparation Guides [35,36]. Briefly, 50 ng of template DNA was used in the initial amplification (PCR1), which was carried out with KAPA HiFi HotStart Ready Mix (Roche AG, Basel, Switzerland) for 25 cycles. PCR products were purified, and 10 ng of the amplicons was used as input for indexing PCRs using the Nextera XT Index v2 Kit (Illumina, San Diego, CA, USA) and KAPA HiFi HotStart Ready Mix, with 8 amplification cycles. Indexed libraries were purified using 0.8 × volume KAPA Pure Beads (Roche AG, Basel, Switzerland). Library quantification was performed with the Equalbit 1 × dsDNA HS Assay Kit (Vazyme Biotech, China) using the same fluorescent plate reader. Library size distribution was evaluated by capillary electrophoresis using a LabChip GX Touch HT Nucleic Acid Analyzer and the DNA NGS 3K Assay Kit (Revvity, Waltham, MA, USA). Libraries were normalized to equimolar concentrations, pooled, and sequenced using 2 × 300 bp paired-end chemistry on the Illumina NextSeq 2000 platform.

### Data analysis

Raw sequencing reads (minimum 100,000 reads per sample) were demultiplexed and adapter-trimmed using the NextSeq Control Software. Low-quality reads (mean quality score <30) were filtered out using the FastQ Toolkit (Illumina, San Diego, CA, USA). Paired-end reads targeting the 16S rRNA and ITS gene regions were processed using the FROGS pipeline [37]. In brief, forward and reverse reads were quality-filtered and merged with VSEARCH [38] using the following parameters: minimum amplicon length of 44 bp, maximum length of 600 bp, and mismatch rate of 0.15. Merged reads were clustered into operational units using SWARM [39], and chimeric sequences were identified and removed with the remove_chimera.py script implemented in FROGS. Singletons and clusters representing fewer than 0.005% of the total read count were excluded from downstream analysis. Taxonomic classification was performed using BLAST [40] against the SILVA 138.2 database [41] for bacterial 16S rRNA sequences and the UNITE Eukaryote 8.2 database [42] for fungal ITS sequences.

Prior to diversity analysis, the ASV (Amplicon Sequence Variant) abundance data were normalized to the sequencing depth of the sample with the lowest read count. For bacterial 16S rRNA datasets, samples were rarefied to 95,742 reads per sample, while fungal ITS datasets were rarefied to 51,602 reads per sample. No samples were excluded due to low read counts. Alpha and beta diversity indices were calculated using the Phyloseq v1.38.0 package within the FROGS framework [43]. Microbial community structure was visualized using multidimensional scaling (MDS). Permutational multivariate analysis of variance (PERMANOVA) was conducted with 9999 permutations to assess the effects of treatments and sampling times on community composition. Differential abundance analysis between treatment groups was performed using the DESeq2 package [44]. In addition, biomarker taxa were identified using linear discriminant analysis (LDA) coupled with the LEfSe algorithm [45]. All other statistical analyses were conducted in R version 4.4.3 [46]. Differences in microbial alpha diversity and the relative abundances of microbial taxa were assessed using one-way analysis of variance (ANOVA), followed by Tukey’s post hoc test for pairwise comparisons. A p-value less than 0.05 was considered statistically significant. Venn diagram analyses were performed using the VennDiagram package, while heatmaps based on relative abundance data were generated using the ComplexHeatmap package. Heatmap values were scaled using the z-score transformation, calculated as z = (x − μ)/σ, where x represents the abundance of a given taxon in a sample, μ is the mean abundance, and σ is the standard deviation across all samples. To explore the associations between environmental and EO variables and microbial alpha diversity, Spearman’s rank correlation was used in R. Redundancy analysis (RDA) was performed to assess how these variables influence microbial community composition. Prior to analysis, microbial abundance datasets were transformed using Hellinger standardization, while soil chemical properties and essential oil variables were z-standardized to remove effects of scale. RDA was then performed in R (vegan package, ordiR2step, 999 permutations). The contribution of soil properties was further validated using the envfit function.

## Results

### Analysis of soil physicochemical properties

The Arany-type plasticity index (MSZ 08–0205:1978) values ranged from 25 to 27 across all samples (25 in *M. spicata* B17, 26 in bulk soil, and 27 in *M. × villosa* B10 and *M. suaveolens* J17), indicating that all soils were classified as sandy loam. Given these narrow differences, and the fact that the *Mentha* cultivars were grown in nearby plots, the texture of both rhizosphere and bulk soils can be considered relatively homogeneous, corresponding to sandy loam texture across all sample types.

Nevertheless, significant differences (p < 0.05) were observed in several chemical parameters between the sample groups (rhizosphere vs. bulk soils, Table 1). The highest nitrogen (nitrite and nitrate) and humus contents were detected in *M.* × *villosa* B10, followed by bulk soil in terms of nitrogen and by *M. suaveolens* J17 in terms of humus. In contrast, *M. spicata* B17 consistently showed the lowest values for both parameters (p < 0.05). Available sodium (Na⁺) levels were highest in *M. suaveolens* J17 and lowest in *M. spicata* B17, with bulk soil and *M.* × *villosa* B10 exhibiting intermediate values. Available phosphorus (P_2_O_5_) and available potassium (K_2_O) concentrations were highest in *M.* × *villosa* B10, while *M. spicata* B17 again had the lowest values. The pH values were similar across all groups, ranging between 7.52 and 7.74, however, despite these small differences, the values in *M. spicata* B17 were significantly lower than in the other samples (p < 0.05).

**Table 1:** Physicochemical properties of soil samples.

	B10	B17	J17	Bulk
**Humus content (%)**	2.30 ± 0.03a	1.50 ± 0.05c	2.29 ± 0.04a	1.90 ± 0.08b
**pH (KCl)**	7.67 ± 0.03a	7.52 ± 0.03b	7.67 ± 0.03a	7.74 ± 0.04a
**(NO**_**3**_ **⁻ + NO**_**2**_ **⁻) (mg/kg)**	18.90 ± 0.16a	3.82 ± 0.08d	11.50 ± 0.10c	12.40 ± 0.13b
**Available Na⁺ (mg/kg)**	66.80 ± 0.15c	52.60 ± 0.11d	71.80 ± 0.09a	68.10 ± 0.09b
**Available P (P**_**2**_ **O**_**5**_ **, mg/kg)**	639.00 ± 16.7a	505.00 ± 9.85c	576.00 ± 20.66b	600.00 ± 18.36ab
**Available K (K**_**2**_ **O, mg/kg)**	268.00 ± 12.33a	167.00 ± 10.70b	187.00 ± 7.21b	187.00 ± 8.66b

Different lowercase letters in the same row indicate significant differences (p < 0.05). Values represent means ± standard deviation (n = 3 per soil type).

Cultivar codes: B10: *M.* × *villosa*, B17: *M. spicata* and J17: *M. suaveolens*.

### Chemical composition of the essential oils

In Table 2 the EO composition analyzed by GC-MS method can be seen in the different mint species. In total, 53 volatile compounds were identified in the distilled essential oils, and results are expressed as relative area percentages. Main compound of *M. spicata* B17 and *M.* × *villosa* B10 was L-carvone (70.93 ± 8.87% and 61.99 ± 1.14%, approximately), while the third species – *M. suaveolens* J17 – contained higher ratios of cis-piperitone-epoxide (63.46 ± 1.04%). *M. spicata* and *M.* × *villosa* were characterized by higher ratios of limonene as well (12.87 ± 1.48% and 15.38 ± 0.71%). Beyond the similarities of these two species some differences can be also highlighted. *M.* × *villosa* B10 contained 1,8-cineol in the ratio of 4.53 ± 0.74, while in the case of *M. spicata* B17 the average was under 1% (0.36 ± 0.10%). On the other hand, *M. spicata* B17 was richer in sesquiterpene compounds – higher ratio was found in the case of ß-caryophyllene (4.08 ± 1.09%), and more sesquiterpenes could be detected. *M. suaveolens* J17 was characterized by a rather different essential oil composition where besides the main compound – cis-piperitone-epoxide – γ-terpinene (9.91 ± 0.25%), piperitenone oxide (5.45 ± 0.19%) and germacrene-D (5.55 ± 0.40%) were detected in higher ratios.

**Table 2:** Composition of essential oils in three *Mentha* cultivars based on GC–MS analysis.

Compound	RT^a^	LRI^b^	A. LRI^c^	B10	B17	J17
α-pinene	5.56	938	932	0.46 ± 0.11	0.30 ± 0.13	0.43 ± 0.13
sabinene	6.52	976	969	0.56 ± 0.06	0.23 ± 0.10	0.42 ± 0.08
ß-pinene	6.64	981	974	0.77 ± 0.08	0.55 ± 0.21	1.33 ± 0.10
3-octanol	6.88	990	982	0.21 ± 0.01	0.38 ± 0.12	0.29 ± 0.08
ß-myrcene	6.99	995	988	1.94 ± 0.12	0.34 ± 0.15	0.40 ± 0.13
α-terpinene	7.79	1018	1014	n.d.	n.d.	0.13 ± 0.02
p-cymene	8.09	1026	1020	n.d.	n.d.	0.12 ± 0.03
**limonene**	8.19	1029	1024	**15.38 ± 0.71**	**12.87 ± 1.48**	**1.37 ± 0.14**
1,8-cineole	8.38	1034	1026	4.53 ± 0.74	0.36 ± 0.10	n.d.
(Z)-ocymene (cis-beta-ocymene)	8.50	1037	1032	0.35 ± 0.02	0.58 ± 0.19	0.26 ± 0.03
(E)-ocymene (trans-beta-ocymene)	8.85	1046	1044	0.12 ± 0.02	0.23 ± 0.01	n.d.
**γ-terpinene**	9.20	1056	1054	**0.16 ± 0.02**	**n.d.**	**9.91 ± 0.25**
trans-sabinene-hydrate	9.73	1070	1098	2.28 ± 0.02	n.d.	n.d.
cis-sabinene-hydrate	10.72	1096	1065	n.d.	n.d.	0.25 ± 0.04
linalool	10.76	1097	1095	0.12 ± 0.01	n.d.	n.d.
octen-3-yl acetate	11.19	1108	1110	n.d.	n.d.	0.24 ± 0.01
trans sabinol	12.51	1140	1137	0.12 ± 0.02	n.d.	n.d.
menthone	13.27	1158	1148	0.40 ± 0.03	n.d.	n.d.
isoborneol	14.26	1182	1179	0.30 ± 0.03	n.d.	0.46 ± 0.04
α-terpineol (cisdehydro)	13.49	1164	1143	0.21 ± 0.01	n.d.	n.d.
terpinene-4-ol	13.96	1175	1174	0.37 ± 0.13	n.d.	0.75 ± 0.12
α-terpineol	14.55	1189	1186	0.22 ± 0.01	n.d.	0.21 ± 0.02
dihydro-carveol	14.68	1192	1192	2.25 ± 0.29	n.d.	n.d.
cis-dihydrocarvone	14.74	1194	1191	1.48 ± 0.16	1.15 ± 0.35	n.d.
trans-dihydrocarvone	15.12	1203	1200	0.07 ± 0.02	0.45 ± 0.22	n.d.
mentha-1,4,8-triene	15.62	1215		0.54 ± 0.04	0.14 ± 0.01	n.d.
hexenyl 2-methyl butanoat (3Z)	16.20	1228	1229	n.d.	0.10 ± 0.01	0.14 ± 0.02
pulegon	16.50	1236	1233	n.d.	n.d.	0.69 ± 0.05
**L-carvone**	16.71	1241	1239	**61.99 ± 1.14**	**70.93 ± 8.87**	**0.05 ± 0.01**
piperitone	17.08	1249	1249	n.d.	0.69 ± 0.03	n.d.
**cis-piperitone-epoxide**	17.42	1257	1250	**n.d.**	**n.d.**	**63.46 ± 1.04**
trans-piperitone-epoxide	17.53	1260	1252	n.d.	n.d.	0.17 ± 0.03
thymol	18.81	1290	1289	n.d.	n.d.	0.51 ± 0.02
dihydro-carveol-acetate (iso)	20.11	1324	1312	0.14 ± 0.04	n.d.	n.d.
**piperitenone oxide**	21.97	1375	1366	**n.d.**	**n.d.**	**5.45 ± 0.19**
ß-bourbonene	22.26	1383	1387	0.67 ± 0.22	1.14 ± 0.44	0.19 ± 0.01
ß-elemene	22.55	1391	1389	n.d.	0.62 ± 0.32	0.19 ± 0.01
cis-jasmone	23.04	1404	1392	n.d.	0.11 ± 0.01	n.d.
α-gurjunene	23.28	1410	1409	n.d.	n.d.	0.10 ± 0.01
ß-caryophyllene	23.68	1420	1417	0.66 ± 0.24	4.08 ± 1.09	3.68 ± 0.16
α-humulene	25.07	1454	1452	0.11 ± 0.03	0.18 ± 0.01	0.44 ± 0.02
ß-farnesene	25.27	1459	1454	n.d.	0.07 ± 0.01	0.32 ± 0.03
alloaromadendrene	25.39	1462	1639	n.d.	0.27 ± 0.01	n.d.
**germacrene-D**	26.18	1482	1480	**1.53 ± 0.69**	**1.48 ± 0.92**	**5.55 ± 0.40**
bicyclogermacrene	26.81	1497	1500	0.36 ± 0.16	0.15 ± 0.01	n.d.
δ-cadinene	27.80	1524	1513	n.d.	0.11 ± 0.01	n.d.
α-cadinene	28.40	1540	1537	n.d.	0.27 ± 0.01	n.d.
ß-cadinene	29.87	1581	1537	n.d.	0.12 ± 0.01	0.12 ± 0.01
spathulenol	29.98	1584	1381	0.13 ± 0.03	0.12 ± 0.01	n.d.
caryophyllene-oxide	30.20	1590	1582	n.d.	0.68 ± 0.01	0.12 ± 0.01
1,10-di-epi-cubenol	31.36	1621	1618	n.d.	0.33 ± 0.01	n.d.
tau-cadinol	32.26	1644	1638	n.d.	0.63 ± 0.39	n.d.
α-cadinol	32.77	1658	1652	n.d.	0.28 ± 0.01	n.d.
Sum:				98.34	98.57	97.71

^a^RT: Retention Time; ^b^LRI: Linear Retention Index; ^c^A. LRI: Adams Linear Retention Index

Values (mean % ± standard deviation, n = 3 per cultivar) represent the relative percentage of each compound within the total oil. Major compounds exceeding 5.00% in each cultivar are highlighted in bold. Cultivar codes: B10: *M.* × *villosa*, B17: *M. spicata* and J17: *M. suaveolens*.

### Base sequence data information

The sequencing dataset included rhizosphere soil samples from three *Mentha* cultivars (*M.* × *villosa* B10, *M. spicata* B17, and *M. suaveolens* J17), as well as bulk soil (Bulk), with three biological replicates per soil type. After quality filtering, a total of 1,519,580 high-quality bacterial sequences were retained and grouped into 3,132 amplicon sequence variants (ASVs). For fungi, 849,925 sequences were clustered into 502 ASVs for downstream analyses. The total ASV counts and Venn analyses reported in this section are based on the full quality-filtered dataset, whereas diversity indices presented in subsequent sections were calculated after rarefaction as described in the Methods.

**Fig 1:**
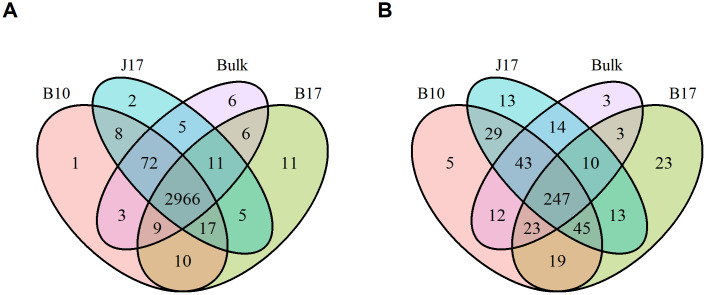
Venn diagrams illustrating the number of unique and shared ASVs identified through NGS for bacterial (A) and fungal (B) communities. Cultivar codes: B10: *M.* × *villosa*, B17: *M. spicata* and J17: *M. suaveolens*.

In the case of bacteria, the number of ASVs per sample ranged from 2,865–3,034 (S2 Table). Samples from *M. spicata* B17 contained significantly (p < 0.05) fewer ASVs compared to the bulk soil and the other two *Mentha* cultivars. As shown in the Venn diagram (Fig 1), 2,966 bacterial ASVs (94.70%) were shared across all sample types, and only a few ASVs were unique to a single soil type. Bulk soil contained six unique ASVs not detected in any rhizosphere samples, while the rhizospheres of the *Mentha* cultivars collectively harbored 54 ASVs absent from the bulk soil. *M. spicata* B17 exhibited the highest number of unique rhizosphere ASVs, despite having the lowest overall ASV count among the samples.

In contrast, fungal communities showed greater variability both among replicates of the same sample type and between different soil types. The number of fungal ASVs per sample ranged from 195 to 352 (S3 Table). Unlike bacteria, bulk soil contained significantly (p < 0.05) fewer fungal ASVs compared to rhizosphere soils. Among the *Mentha* samples, the highest average ASV count was observed in *M.* × *villosa* (B10; 339.67), and the lowest in *M. spicata* (B17; 287.00). A total of 247 fungal ASVs (49.20%) were shared among all sample types. Only three ASVs were found exclusively in the bulk soil, whereas 147 ASVs were unique to the rhizospheres of the *Mentha* cultivars. Notably, all 34 detected Glomeromycota ASVs, representing potential arbuscular mycorrhizal fungi, were exclusively present in the rhizosphere soils and absent from the bulk soil. Collectively, the ASV-level comparisons indicate that *M. spicata* B17 differs from the other two cultivars in overall richness and in the distribution of unique ASVs. Alpha and beta diversity metrics and differential abundance analysis were assessed and are discussed in the following sections.

### Bacterial Community Composition and Diversity

**Fig 2:**
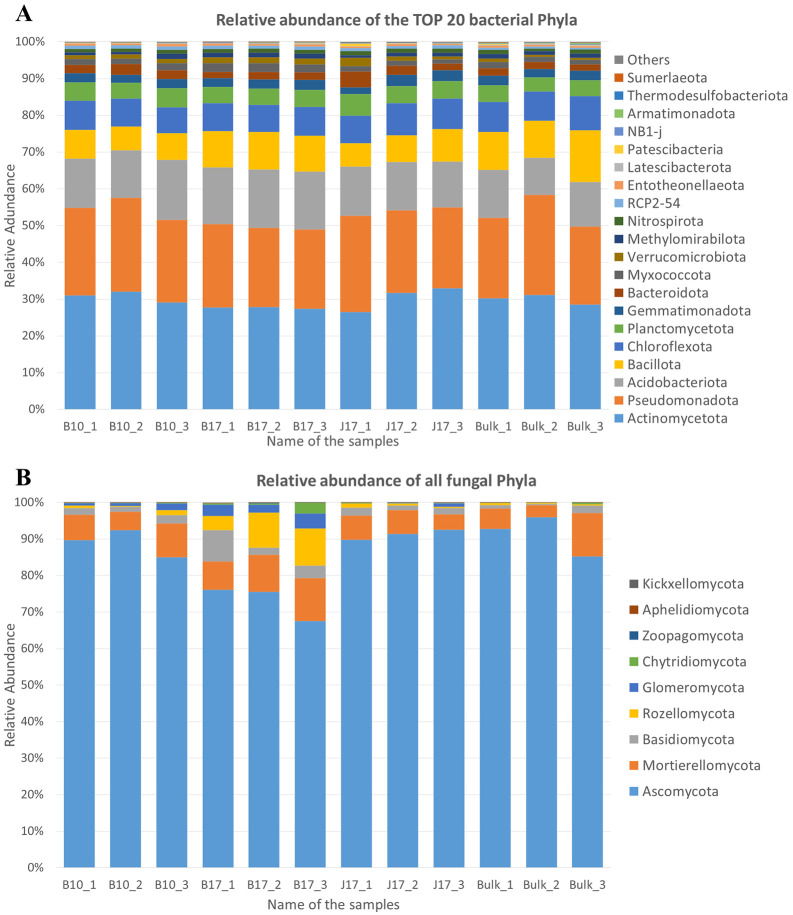
Relative abundance of bacterial (A) and fungal (B) communities at phylum level. Cultivar codes: B10: *M.* × *villosa*, B17: *M. spicata* and J17: *M. suaveolens*.

**Fig 3:**
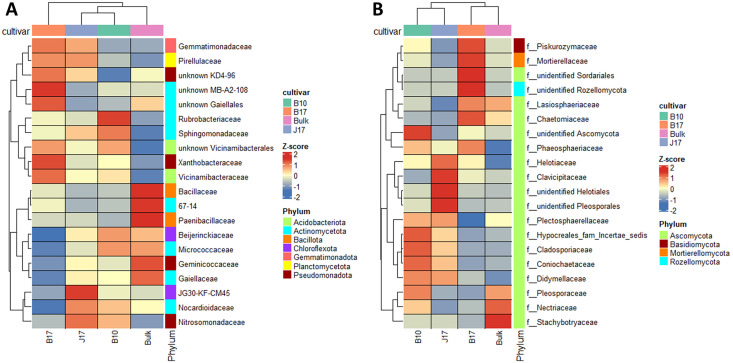
Heatmap of the 20 most abundant bacterial (A) and fungal (B) families in the soil samples. The color scale represents the Z-scores of the relative abundance values for each family within each cultivar. Cultivar codes: B10: *M.* × *villosa*, B17: *M. spicata* and J17: *M. suaveolens*. n = 3 biological replicates per soil type (each *Mentha* cultivar and bulk soil).

A total of 28 phyla, 70 classes, 144 orders, 214 families, 449 genera and 646 species were detected by sequencing (S2 Table). The five most abundant bacterial phyla across all samples were Actinomycetota, Pseudomonadota, Acidobacteriota, Bacillota, and Chloroflexota, with average relative abundances of 29.67%, 23.23%, 13.64%, 9.05%, and 7.92%, respectively (Fig 2a). Only seven additional phyla exhibited average abundances above 1%, while the remaining 16 identified phyla collectively accounted for just 1.99% of the total bacterial community. Notable differences in the relative abundance of specific bacterial taxa were observed among the different soil types. For instance, the phylum Bacillota was significantly (p < 0.05) more abundant in bulk soil (11.46 ± 2.22%) compared to the rhizosphere samples (8.25 ± 1.46%). Significant compositional differences were also found at lower taxonomic levels. Family-level differences are visualized in the heatmap presented in Fig 3a and are further detailed in the differential abundance analyses.

**Fig 4:**
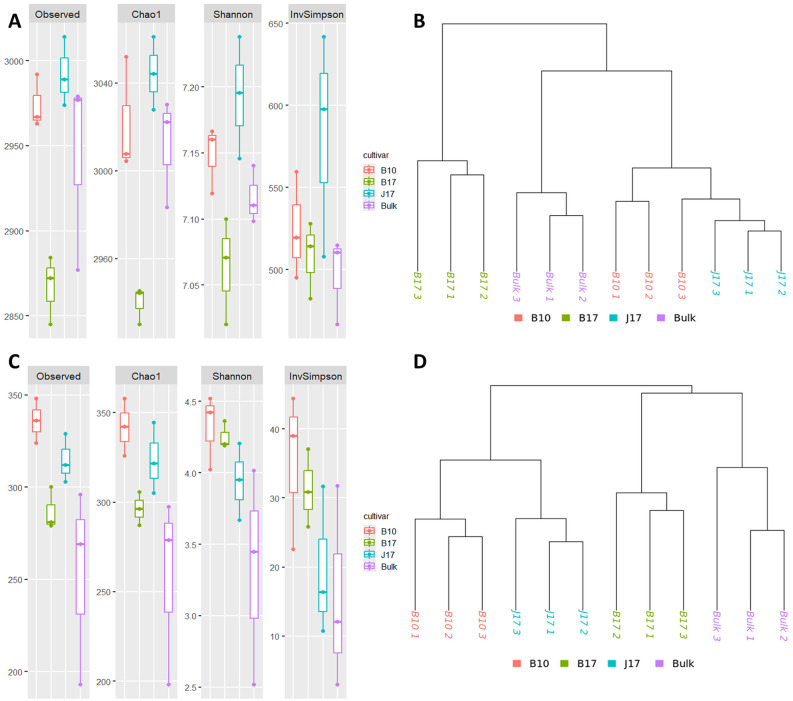
Microbial diversity. Alpha diversity indices of the rhizosphere soil samples grouped by cultivars in the case of bacteria (A) and fungi (C). Beta diversity patterns based on hierarchical clustering using unweighted UniFrac distance matrices are shown for bacterial (B) and fungal (D) communities. Clustering was performed using Ward’s minimum variance method (ward.D2 linkage). Cultivar codes: B10: *M.* × *villosa*, B17: *M. spicata* and J17: *M. suaveolens*. n = 3 biological replicates per soil type (each *Mentha* cultivar and bulk soil).

The analysis of bacterial alpha diversity revealed significant differences between sample types (Fig 4a). Statistically significant differences were found in the number of observed species (p < 0.01), as well as in the Chao1 (p < 0.01) and Shannon indices (p < 0.05). In all three metrics, the *M. spicata* B17 rhizosphere samples exhibited the lowest diversity, while the *M. suaveolens* J17 samples showed the highest values. Although the Inverse Simpson index did not show a statistically significant difference, *M. suaveolens* J17 samples still yielded the highest values, whereas the other three groups (*M. spicata* B17, *M.* × *villosa* B10, and bulk) had similar values.

The beta-diversity analysis also confirmed differences between the samples. The hierarchical clustering based on unweighted UniFrac distances revealed two major clusters: one comprising the *M. suaveolens* J17 and *M.* × *villosa* B10 rhizosphere samples, and another that included the *M. spicata* B17 samples as a separate subgroup, clearly distant from the others (Fig 4b). The bulk soil samples formed a more loosely connected subgroup, indicating intermediate similarity between some rhizosphere communities and the non-rhizosphere environment. This finding aligns with the alpha diversity results where *M. spicata* B17 exhibited the lowest diversity. PERMANOVA analysis based on unweighted UniFrac distances also indicated that cultivar identity was a strong determinant of bacterial community structure, explaining 69.29% of the variation (p < 0.001).

### Fungal Community Composition and Diversity

A total of 9 phyla, 24 classes, 50 orders, 96 families, 162 genera and 220 species were detected through sequencing (S2 Table). Among the identified fungal phyla, Ascomycota (87.44%), Mortierellomycota (7.09%), Basidiomycota (2.11%), and Rozellomycota (1.95%) exhibited average relative abundances exceeding 1% across all samples (Fig 2b). Glomeromycota showed an average relative abundance of 0.98% across all samples; however, it was completely absent in bulk soil samples, while present in rhizosphere soils at relative abundances ranging from 0.05% to 4.10%. Four additional fungal phyla were identified in the soil samples, but their combined relative abundance did not exceed 0.5%. Several ASVs could not be confidently assigned even at the phylum level and were therefore excluded from subsequent analyses.

Fungal taxonomic composition showed high variability, both between different soil types and among biological replicates within the same group. The mean relative abundance of Ascomycota was 73.10% ± 4.79% in *M. spicata* B17 samples, significantly lower (p < 0.01) than in *M.* × *villosa* B10 (89.07% ± 3.72%), *M. suaveolens* J17 (91.22% ± 1.39%), and bulk soil (91.35% ± 5.48%). Interestingly, while the relative abundance of *Ascomycota* was significantly lower in the rhizosphere of *M. spicata* B17, the cumulative abundance of non-Ascomycota fungal phyla tended to be higher in these samples. For example, *Mortierellomycota, Rozellomycota,* and *Glomeromycota* all were more abundant in *M. spicata* B17 compared to the other cultivars and the bulk soil, suggesting a shift in fungal community structure associated with this cultivar. Pronounced differences in fungal community composition were also evident at the family level. These variations are illustrated in the heatmap shown in Fig 3b, and are further explored through differential abundance analyses described below.

Analysis of fungal alpha diversity revealed significant differences (p < 0.05) in the number of observed species, Chao1, and Shannon indices, similarly to the bacterial community. However, in contrast to the bacterial results, where *M. spicata* B17 showed the lowest values, the bulk soil samples exhibited the lowest fungal alpha diversity (Fig 4c). Regarding the Inverse Simpson index, both bulk soil and *M. suaveolens* J17 samples displayed lower values compared to the other two cultivars, although these differences were not statistically significant.

The beta-diversity analysis confirmed differences in fungal community composition between sample types. Hierarchical clustering based on unweighted UniFrac distances (Ward.D2 linkage) revealed two distinct clusters: one comprising the rhizosphere samples of *M. suaveolens* J17 and *M. × villosa* B10, and another including *M. spicata* B17 and bulk soil samples (Fig 4d). The close association of B17 and bulk soil suggests a divergence of the B17 fungal community from the other rhizosphere types. PERMANOVA analysis based on unweighted UniFrac distances further confirmed that cultivar identity significantly influenced fungal community structure, explaining 60.85% of the variation (p < 0.001).

### Discriminative taxa driving differences between rhizosphere and bulk soil

**Fig 5:**
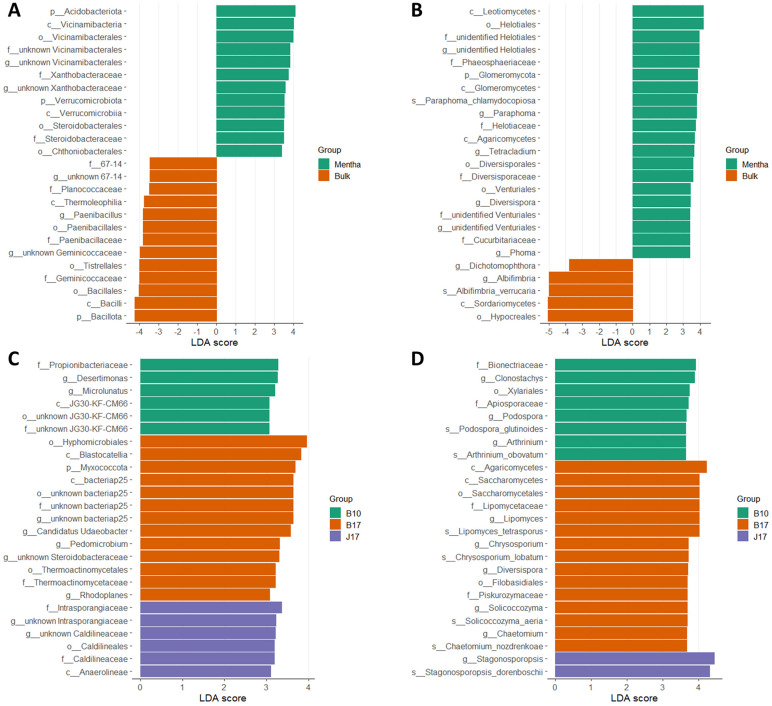
Top 25 differentially abundant taxa identified by LEfSe analysis. Panels A and B show the comparison between bulk soil and *Mentha* rhizosphere samples, based on merged data from all cultivars. Panel A displays differentially abundant bacterial taxa, while panel B presents fungal taxa. Panels C and D illustrate taxa with significant differential abundance among the rhizosphere samples of the three *Mentha* cultivars, excluding bulk soil. Panel C shows bacterial taxa, and panel D fungal taxa. Cultivar codes: B10: *M.* × *villosa*, B17: *M. spicata* and J17: *M. suaveolens*. n = 3 biological replicates per soil type (each *Mentha* cultivar and bulk soil).

Differential abundance analysis using DESeq2 identified 110 ASVs that were significantly more abundant in bulk soil compared to the rhizosphere (padj < 0.05), while 225 ASVs were significantly enriched in the rhizosphere (S1 Fig). However, many of these differentially abundant ASVs had low mean sequence counts. Therefore, additional biomarker identification was performed using LEfSe. Both DESeq2 and LEfSe analyses consistently highlighted the phylum Bacillota as significantly more abundant in bulk soil (bulk: 11.46 ± 2.22%, rhizosphere: 8.25 ± 2.22%), along with associated taxa such as the class Bacilli, the order Bacillales, and the families *Planococcaceae* and *Paenibacillaceae* (Fig 5a). Additional bulk-specific features included members of the phylum Pseudomonadota, particularly the order Tistrellales and the family *Geminicoccaceae*, including an unidentified genus within this family. The class Thermoleophilia and family 67−14, belonging to the phylum Actinomycetota, was also more abundant in bulk soil according to both methods.

A substantial proportion of ASVs significantly enriched in rhizosphere soils belonged to the phyla Pseudomonadota (52 ASVs) and Acidobacteriota (49 ASVs), according to DESeq2 analysis. These findings were in line with the LEfSe results, which identified Acidobacteriota, particularly the class Vicinamibacteria and the order Vicinamibacterales (including several unclassified families and genera), as biomarkers characteristic of rhizosphere samples. LEfSe also confirmed that several taxa within the Pseudomonadota phylum, including the family *Xanthobacteraceae* and the order Steroidobacterales, were enriched in rhizosphere soils. Although only nine significantly different ASVs belonged to the phylum Verrucomicrobiota, this phylum and its associated taxa (Verrucomicrobiia, Chthoniobacterales) were also identified as rhizosphere-specific features.

Out of the 502 fungal ASVs detected, 7 were significantly more abundant in bulk soil and 31 in rhizosphere soils (padj < 0.05, DESeq2, S1 Fig). Among the bulk-associated taxa, *Albifimbria verrucaria* emerged as a particularly strong biomarker. Five of the seven bulk-enriched ASVs were affiliated with this species. LEfSe analysis (Fig 5b) corroborated this result, ranking *Albifimbria* among the top five bulk-specific biomarkers, with an LDA score exceeding 5. This pattern was primarily driven by a single bulk replicate, where *Albifimbria* reached a relative abundance of 58.58%, while its abundance remained low in the other bulk samples (2.11% and 2.90%) and across the rhizosphere samples (7.90% in one of the B10 replicates, and ranging from 0.12% to 1.55% in all others). Another bulk soil-associated biomarker was the genus *Dichotomophthora*, which was consistently detected in all three bulk soil samples, whereas it was present in only one of the rhizosphere samples.

Rhizosphere-specific Ascomycota biomarkers included the genera *Phoma* and *Paraphoma*, as well as members of the orders Venturiales and Helotiales, represented by several unclassified families and genera, along with *Tetracladium* from the family *Helotiaceae*. As previously mentioned, Glomeromycota sequences were exclusively detected in rhizosphere soils, and accordingly, taxa such as Glomeromycetes, Diversisporales, *Diversisporaceae*, and *Diversispora* were also identified as strong rhizosphere-associated biomarkers.

### Comparison of the rhizosphere microbiota among *Mentha* cultivars

To investigate differentially abundant microbial taxa among the three *Mentha* cultivars, we excluded bulk soil samples and focused exclusively on rhizosphere communities. Pairwise comparisons using DESeq2 revealed that *M.* × *villosa* B10 and *M. suaveolens* J17 had the most similar bacterial profiles, with only 85 ASVs (2.71%) differing significantly, 41 were more abundant in J17, and 44 in B10. In contrast, comparisons involving *M. spicata* B17 showed far greater differences: 1,190 (37.99%) ASVs differed significantly between *M. spicata* B17 and *M.* × *villosa* B10, and 1,282 (40.93%) between *M. spicata* B17 and *M. suaveolens* J17 (S2 Fig).

LEfSe analysis (Fig 5c) identified distinct biomarkers for each cultivar. In *M. suaveolens* J17, significant indicators included members of the phylum Chloroflexota (class Anaerolineae, order Caldilineales, family *Caldilineaceae*) and the family *Intrasporangiaceae* within Actinomycetota. The *M.* × *villosa* B10 rhizosphere was characterized by an uncultured Chloroflexota lineage (JG30-KF-CM66) and two Actinomycetota-associated taxa: the genus *Desertimonas* and the family *Propionibacteriaceae*, including the genus *Microlunatus*. The *M. spicata* B17 rhizosphere harbored the highest number of discriminative features, including members of Myxococcota (bacteriap25 group), Acidobacteriota (class Blastocatellia), and Pseudomonadota (order Hyphomicrobiales, genera *Pedomicrobium* and *Rhodoplanes*, and an unclassified *Steroidobacteraceae*). Additional biomarkers of *M. spicata* B17 included the order Thermoactinomycetales and the family *Thermoactinomycetaceae* (Bacillota), as well as *Candidatus Udaeobacter* (Verrucomicrobiota).

The fungal communities showed a similar pattern in the DESeq2 analysis (S2 Fig). Of the 502 fungal ASVs, only 33 (6.57%) differed significantly between *M.* × *villosa* B10 and *M. suaveolens* J17, while 84 (16.73%) and 112 (22.31%) ASVs differed between *M. spicata* B17 and *M.* × *villosa* B10, and *M. spicata* B17 and *M. suaveolens* J17, respectively. LEfSe analysis (Fig 5d) identified *Stagonosporopsis* as a prominent biomarker for *M. suaveolens* J17, with LDA scores exceeding 4. For *M.* × *villosa* B10, notable fungal biomarkers included the order Xylariales and its associated family *Apiosporaceae*, genus *Arthrinium* and species *A. obovatum,* as well as the genus *Podospora* (*P. glutinoides*), and the genus *Clonostachys* (*Bionectriaceae*). In line with bacterial data, the *M. spicata* B17 rhizosphere also exhibited the most distinct fungal profile. *Lipomyces tetrasporus* (class Saccharomycetes, order Saccharomycetales, family *Lipomycetaceae*) was represented by a single ASV present in all samples, but showed significantly higher mean relative abundance in *M. spicata* B17 (2.17%) compared to *M.* × *villosa* B10 (0.19%) and *M. suaveolens* J17 (0.05%) (p < 0.001), and was therefore identified as a robust biomarker for *M. spicata* B17. Similarly, *Solicoccozyma aeria* (order Filobasidiales, family *Piskurozymaceae*, genus *Solicoccozyma*) was also represented by one ASV, with significantly higher relative abundance in *M. spicata* B17 (1.68%) than in *M.* × *villosa* B10 (1.21%) and *M. suaveolens* J17 (0.84%) (p < 0.05), although the magnitude of difference was smaller than that of *L. tetrasporus*. Other characteristic fungal biomarkers of *M.* *spicata* B17 included *Diversispora* (Glomeromycota), *Chrysosporium lobatum* and *Chaetomium nozdrenkoae* (Ascomycota), and members of the class Agaricomycetes (Basidiomycota).

### Comparison of Glomeromycota abundance in soil and root mycorrhizal colonization

All root samples exhibited clear evidence of AMF colonization, with colonization rates ranging from 56.8% to 78.4%. The lowest colonization rate was observed in *M. suaveolens* (J17, 56.8 ± 5.6%) while the highest was recorded in *M. spicata* (B17, 78.4 ± 8.0%). An intermediate level of colonization was observed in *M. × villosa* B10 at a rate of 61.6 ± 5.4%. Despite some variation in staining intensity and distribution among replicates, microscopic examination consistently revealed the presence of all major AMF structures (hyphae, arbuscules, and vesicles) across all three cultivars, indicating the establishment of functional symbiosis.

**Fig 6:**
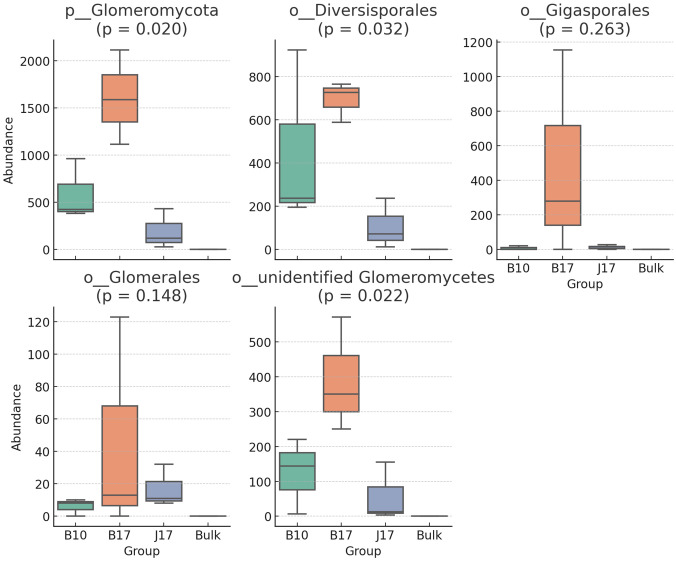
Abundance of dominant Glomeromycota taxa in the rhizosphere and bulk soil samples. The figure shows the distribution of normalized abundance values (sequence counts) for five representative Glomeromycota taxa across the rhizosphere soils of three *Mentha* cultivars (B10: *M. × villosa*, B17: *M. spicata*, J17: *M. suaveolens*) and bulk soil. Statistical differences were assessed using the Kruskal–Wallis test, and the resulting p-values are indicated above each panel. n = 3 biological replicates per soil type (each *Mentha* cultivar and bulk soil).

These findings were in line with the high-throughput sequencing results, which similarly suggested enhanced AMF activity in *M. spicata* rhizospheres. Sequences assigned to the phylum Glomeromycota, which includes AMF, were exclusively detected in rhizosphere soil samples and completely absent from bulk soil. A total of 34 Glomeromycota ASVs were identified, classified into four main groups: 19 ASVs affiliated with the *Diversisporaceae* family, 6 with *Gigasporaceae*, 2 with *Glomeraceae*, and 7 ASVs assigned only at the order level (unidentified Glomeromycetes). As shown in Fig 6, significantly higher (p < 0.05) Glomeromycota sequence counts were detected in the rhizosphere of *M. spicata* B17 compared to the other soil types. This pattern was also reflected at the family level, where *M. spicata* B17 samples harbored the highest number of sequences across all families, except for *Diversisporaceae*, in which *M. × villosa* B10 samples exhibited comparable counts (relative abundance in *M. × villosa* B10: 0.88 ± 0.79%, *M. spicata* B17: 1.35 ± 0.18%).

Overall, the average relative abundance of Glomeromycota in rhizosphere soils followed the trend: *M. spicata* B17 (3.11 ± 0.96%) > *M. × villosa* B10 (1.14 ± 0.63%) > *M. suaveolens* J17 (0.37 ± 0.07%), mirroring the patterns observed in root colonization rates.

### Associations between microbial diversity, community structure, and soil physicochemical properties

Correlation analysis between bacterial and fungal alpha diversity indices and soil properties revealed distinct patterns (Table 3). Humus content showed the strongest and most consistent positive correlations with richness-related indices (Observed species and Chao1) for both bacteria and fungi (bacterial Observed species: ρ = 0.709, p < 0.05; bacterial Chao1: ρ = 0.744, p < 0.05; fungal Observed species: ρ = 0.726, p < 0.01; fungal Chao1: ρ = 0.688, p < 0.05). In bacteria, Na⁺ concentration also correlated significantly and positively with observed species, Chao1, and Shannon indices, suggesting a potential stimulatory effect on richness and diversity. In contrast, Na⁺ and pH exhibited weak negative correlations with fungal Shannon and Inverse Simpson indices, though not statistically significant, potentially indicating sensitivity of fungal evenness to salinity and alkalinity. Additionally, available potassium (K) showed a moderate, significant positive correlation with fungal richness (observed species index).

**Table 3:** Correlation between alpha diversity indices of rhizosphere bacteria and fungi and selected soil parameters.

	Diversity index	Humus	Available K	Available Na⁺	N (NO3⁻ + NO2⁻)	pH	Available P
**Bacteria**	**Observed**	0.709*	0.378	0.741*	0.462	0.495	0.399
	**Chao1**	0.744*	0.441	0.769*	0.448	0.526	0.413
	**Shannon**	0.775*	0.427	0.608*	0.385	0.288	0.357
	**InvSimpson**	0.263	−0.070	0.140	−0.063	−0.063	−0.154
**Fungi**	**Observed**	0.726**	0.629*	0.112	0.420	−0.270	0.427
	**Chao1**	0.688*	0.566	0.098	0.371	−0.323	0.357
	**Shannon**	0.091	0.217	−0.517	0.021	−0.421	0.084
	**InvSimpson**	0.074	0.301	−0.441	0.133	−0.211	0.231

Values are Spearman’s rank correlation coefficients (ρ) between alpha diversity indices (Observed species, Chao1, Shannon, and Inverse Simpson) and soil chemical properties (humus content, available K and Na ⁺ , nitrate + nitrite nitrogen, pH, and available P). Asterisks indicate significant correlations (*p < 0.05, **p < 0.01).

Cultivar codes: B10: *M.* × *villosa*, B17: *M. spicata* and J17: *M. suaveolens*.

**Fig 7:**
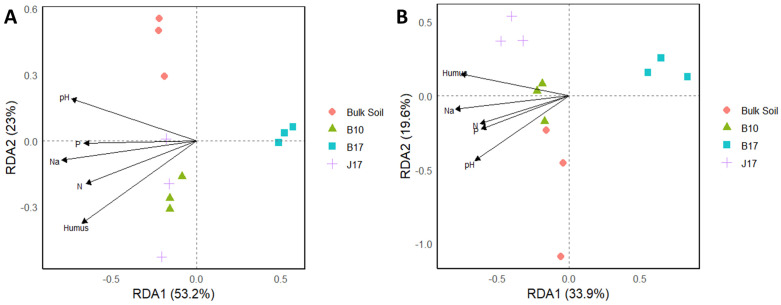
Redundancy analysis of bacterial (A) and fungal (B) communities in relation to soil properties. Abbreviation of soil chemical properties: humus: humus content; Na: available Na ⁺ ; N: nitrate + nitrite nitrogen; pH; and P: available P. Cultivar codes: B10: *M.* × *villosa*, B17: *M. spicata* and J17: *M. suaveolens*. RDA1 and RDA2 together explained 76.2% of the variance in bacterial communities (53.2% and 23.0%, respectively) and 53.5% in fungal communities (33.9% and 19.6%, respectively). Predictors showed moderate multicollinearity; therefore, ordination axes are interpreted as representing combined environmental gradients rather than independent effects of individual variables. n = 3 biological replicates per soil type (each *Mentha* cultivar and bulk soil).

While correlation analysis highlighted associations between individual diversity metrics and soil variables, we next employed redundancy analysis (RDA) to assess how environmental factors influence overall microbial community composition. For bacterial communities, the RDA model was highly significant (R^2^ = 0.762, Adj. R^2^ = 0.526, p = 0.001), with the first two axes (RDA1 and RDA2) explaining 53.2% and 23.0% of the constrained variance, respectively (Fig 7a). In total, the soil variables accounted for 76.2% of the variation in bacterial community composition. The ordination revealed clear separation of samples by soil type, with bacterial assemblages primarily structured by humus content, phosphorus, and potassium, while nitrogen, sodium, and pH exerted no significant effects. Permutation tests confirmed humus (F = 7.57, p = 0.001), phosphorus (F = 3.14, p = 0.016), and potassium (F = 3.74, p = 0.012) as significant explanatory variables. In contrast, pH (F = 1.45, p = 0.201), nitrogen (F = 1.13, p = 0.340), and sodium (F = 1.20, p = 0.292) were not significant contributors to bacterial community variation.

High variance inflation factors (VIF > 10) were observed among variables, indicating strong multicollinearity. All predictors were nevertheless retained in the model to capture the overall influence of soil chemistry, with observed gradients likely reflecting combined rather than isolated variable effects.

Fungal communities showed a similar but slightly weaker response to soil chemistry (Fig 7b). The fungal RDA model explained 53.5% of the total variation, with RDA1 (33.9%) aligned primarily with humus and RDA2 (19.6%) reflecting gradients in phosphorus and potassium. The overall model was significant (R^2^ = 0.535, Adj. R^2^ = 0.318, p = 0.001), and ordination plots again showed separation among soil groups, though fungal communities were compositionally more stable across sites compared to bacteria. Permutation tests identified humus (F = 3.51, p = 0.001), phosphorus (F = 1.78, p = 0.021), and potassium (F = 2.00, p = 0.012) as significant predictors of fungal community structure, with pH showing marginal significance (F = 1.56, p = 0.051). Nitrogen (F = 0.97, p = 0.340) and sodium (F = 1.30, p = 0.292) did not contribute significantly. Overall, rhizosphere soils harbored distinct bacterial and fungal assemblages compared to bulk soil, reflecting host-associated differences in community composition.

### Associations between microbial community structure and essential oil composition

To further explore the potential associations between plant chemotype and patterns of microbial diversity, we examined correlations with dominant secondary metabolites of the host plants (Table 4). Spearman’s rank correlation analysis revealed significant associations between bacterial alpha diversity and specific EO constituents characteristic of the respective *Mentha* cultivars. Interestingly, the most prominent correlations were observed not for the major EO components (e.g., carvone, limonene), but for minor constituents such as γ-terpinene, which was nearly exclusive to the *Mentha suaveolens* J17 samples. Despite its relatively low overall abundance, γ-terpinene exhibited strong positive correlations with several bacterial diversity indices, including observed species (ρ = 0.898, p < 0.01), Chao1 (ρ = 0.898, p < 0.01), and Shannon (ρ = 0.746, p < 0.05), indicating a strong cultivar-associated relationship with bacterial richness and diversity metrics. In contrast, L-carvone, a dominant compound in both *M.* × *villosa* B10 and *M. spicata* B17, showed a significant negative correlation with the Shannon index (ρ = –0.817, p < 0.05), reflecting an inverse association between carvone-dominated chemotypes and bacterial evenness.

**Table 4:** Correlation between alpha diversity indices of rhizosphere bacteria and fungi and major essential oil components.

	Diversity index	limonene	L-carvone	piperitenone oxide	germacrene-D	γ-terpinene	cis-piperitone-epoxide
**Bacteria**	**Observed**	−0.183	−0.633	0.644	0.617	0.898**	0.644
	**Chao1**	−0.200	−0.667	0.644	0.583	0.898**	0.644
	**Shannon**	−0.333	−0.817*	0.564	0.417	0.746*	0.564
	**InvSimpson**	−0.483	−0.617	0.327	0.067	0.322	0.327
**Fungi**	**Observed**	0.300	−0.500	0.109	0.033	0.475	0.109
	**Chao1**	0.209	−0.527	0.164	0.000	0.485	0.164
	**Shannon**	0.550	0.433	−0.614	−0.383	−0.407	−0.614
	**InvSimpson**	0.550	0.433	−0.614	−0.383	−0.407	−0.614

Values are Spearman’s rank correlation coefficients (ρ) between alpha diversity indices (Observed species, Chao1, Shannon, and Inverse Simpson) and EO variables (limonene, L-carvone, piperitenone oxide, germacrene-D, γ-terpinene, piperitone-epoxide). Asterisks indicate significant correlations (*p < 0.05, **p < 0.01). Only major essential oil components with an average relative abundance exceeding 5% in at least one cultivar were included in the correlation analysis.

Cultivar codes: B10: *M.* × *villosa*, B17: *M. spicata* and J17: *M. suaveolens*.

For fungal alpha diversity, no statistically significant correlations were detected with EO compounds (p > 0.05). However, certain compositional trends were observed. Limonene and L-carvone showed weak-to-moderate positive correlations with fungal richness and diversity indices, whereas piperitenone oxide and cis-piperitone epoxide tended to correlate negatively with Shannon and Inverse Simpson indices.

**Fig 8:**
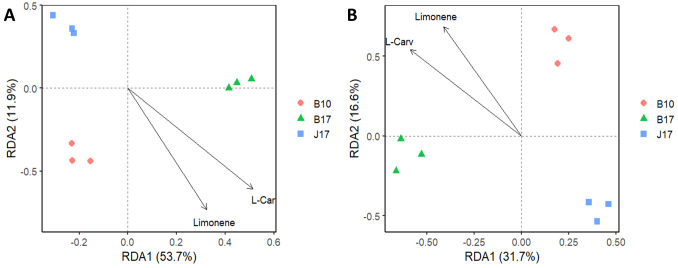
Redundancy analysis of bacterial (A) and fungal (B) communities in relation to major essential oil components. Only major essential oil components with an average relative abundance exceeding 5% in at least one cultivar were included in the correlation analysis. Cultivar codes: B10: *M.* × *villosa*, B17: *M. spicata* and J17: *M. suaveolens*. RDA1 and RDA2 explained 65.6% of the variance in bacterial communities (53.7% and 11.9%, respectively) and 48.3% in fungal communities (31.7% and 16.6%, respectively). Variance inflation factors (VIF > 10) indicated multicollinearity among predictors; thus, axes are interpreted as reflecting combined chemotype-associated gradients rather than independent effects of individual compounds. n = 3 biological replicates per cultivar.

While pairwise correlation analysis revealed significant associations between individual alpha diversity indices and specific major EO components, RDA was employed to further assess the extent to which EO components were statistically associated with variation in overall microbial community composition. For bacterial communities, the constrained RDA model was statistically significant (R^2^ = 0.656, Adj. R^2^ = 0.541, p = 0.004), and the stepwise model selection procedure identified L-carvone and limonene as key explanatory variables. Together, these two compounds accounted for 65.6% of the total variance in bacterial community structure. Both predictors were individually significant based on permutation tests (L-carvone: F = 5.19, p = 0.009; limonene: F = 6.22, p = 0.018). Although variance inflation factors (VIF = 12.6 for both compounds) indicated multicollinearity, the combined model remained robust and captured the dominant EO-related gradients. In this context, the RDA axes were interpreted as reflecting combined environmental gradients rather than independent effects of individual predictors. Correlated variables were retained to represent the overall chemical context of each cultivar for descriptive purposes. The ordination plot showed that vectors for L-carvone and limonene were oriented toward the *M.* × *villosa* B10 samples, with *M. spicata* B17 samples clustering nearby. In contrast, *M. suaveolens* J17 samples separated distinctly along the RDA axes, consistent with their contrasting chemotype profile (Fig 8a).

Fungal communities showed a similar, but weaker response to EO composition. The reduced RDA model again identified L-carvone and limonene as the most informative variables, jointly explaining 48.3% of the variance in fungal community structure across the first two RDA axes (RDA1: 31.7%; RDA2: 16.6%; R^2^ = 0.483, Adj. R^2^ = 0.310, p = 0.006). Both compounds were significant in permutation tests (L-carvone: F = 2.30, p = 0.012; limonene: F = 2.73, p = 0.006), and the overall model was also significant. The ordination of the ITS dataset closely mirrored that of the 16S dataset, with vectors for L-carvone and limonene pointing toward *M.* × *villosa* B10 samples and away from *M. spicata* B17 and *M. suaveolens* J17 (Fig 8b). These ordination patterns therefore reflect chemotype-associated community differentiation rather than direct measurements of rhizosphere chemical exposure.

## Discussion

### Plant-microbe interactions and ecological context in *Mentha* rhizospheres

The rhizosphere is a biologically active soil compartment where plant genotype and chemistry jointly shape the structure and function of microbial communities. In aromatic and medicinal plants, this interaction is further influenced by the production of secondary metabolites and EOs that can serve as signaling molecules or selective antimicrobial agents, thereby modulating microbial colonization patterns [7,14,47]. Members of the *Lamiaceae* family, such as *Mentha*, *Thymus*, and *Salvia*, are particularly notable for their ability to release volatile and phenolic compounds through both roots and above-ground tissues, resulting in pronounced rhizosphere effects and distinct soil microbial assemblages [26,48–50]. In *Mentha* species, recent studies demonstrated that mints can alter the composition of bacterial and fungal communities in their rhizospheres through the continuous release of EO constituents and other specialized metabolites [17]. Similarly, microbial inoculation experiments have shown that interactions between soil bacteria and *Mentha* roots can further enhance EO biosynthesis, underscoring the bidirectional nature of plant-microbe relationships in these aromatic systems [48,51]. Given this close biochemical connection between *Mentha* plants and their rhizosphere, examining the chemical composition of their EOs provides a crucial starting point for understanding potential drivers of microbial differentiation. The three taxa studied here – *M. × villosa*, *M. spicata*, and *M. suaveolens* – share a close genetic relationship. *M. spicata* is an ancient natural hybrid of *M. longifolia* and *M. suaveolens*, while *M. × villosa* is a hybrid of *M. spicata* and *M. suaveolens*. Their comparison under similar field conditions thus offers valuable insights into how subtle genetic and metabolic differences may influence both EO profiles and the associated rhizosphere microbiota.

### EO composition of the three *Mentha* species

To better understand the biochemical factors that may shape rhizosphere processes, the EO composition of the studied *Mentha* species was examined to provide an overview of their metabolic diversity. Their EO composition was largely consistent with previous literature [52]. Usually, *Mentha* taxa are rich in monoterpenes originating from the biosynthesis via α-terpinyl cation, and its oxidation processes. Limonene-oxo products are often accompanied by highly variable quantities of 1,8-cineole (another α-terpinyl metabolite widespread in many, non-related plant taxa) and certain sesquiterpenes: germacrene D and/or β-caryophyllene. Although there are some peculiar chemotypes, even cultivars of *M. spicata* performing other pathways (reviewed by [19]), the typical EO from aerial parts of *M. spicata, M. suaveolens*, and *M.* × *villosa* is provided by the 2-oxo or 3-oxo branch of the limonene pathway, resulting in the carvone class or the menthone class of monoterpenes, respectively.

*M. spicata* B17 is rich in L-carvone and residual limonene. Even if the unique chemodiversity of *M. spicata* is known [19], in most cases, L-carvone was identified as the main constituent [53–55]. Its ratio can vary between 29–85.41% [56,57]. In our research the analyzed *M. spicata* B17 sample contained L-carvone in the ratio of 70.93 ± 8.87%. Second main compound was limonene (12.87 ± 1.48%) – that result was also in accordance with the previous results, where the area percentages were found between 6.13% [58] and 28.10% [59].

In the case of *M. × villosa* the EO composition can be connected to the geographical origin [19]. European originated samples are usually rich in L-carvone and residual limonene, while the Brazilian *M. × villosa* are almost free from this compound [60]. In the South American populations the main EO compound is piperitenone epoxide [61,62]. In our sample L-carvone was the most characteristic volatile compound with the ratio of 61.99 ± 1.14%, therefore it can be grouped into the European type of *M. × villosa*. Regarding sesquiterpenes, germacrene-D was the one in the highest ratio (1.53 ± 0.69%), which may be considered as relatively low.

*M. suaveolens* was characterized by rather different EO composition compared to *M. spicata* and *M. × villosa*. According to the literature, *M. suaveolens* exhibits at least two major chemotypes: one dominated by piperitone/piperitenone epoxide derivatives [52,63,64], and another by pulegone [63,65,66]. *M. suaveolens* J17 demonstrates a limonene-3-oxo epoxide type, where these epoxides are accompanied by low amounts of their relative ketones like pulegone (0.69 ± 0.05%) and, unconventionally, by ca. 10 area% of γ-terpinene. This latter is not typical in *Mentha* spp. as a major constituent. However, no γ-terpinene-oxo products (thymol isomers, cymene isomers, or cymen-8-ol, etc.) were detected. In the *M. suaveolens* J17 sample, the main compound was cis-piperitone epoxide (63.46 ± 1.04%), and piperitenone oxide (5.45 ± 0.19%). This pattern is in line with the previous findings [52,63,64]. Additionally, *M. suaveolens* J17 does not contain a high ratio of residual limonene, which is a typical trait of limonene-3-oxo type mints [67]. Among the sesquiterpene constituents, germacrene D is the leading one (5.55 ± 0.40%). Other studies reported it from *M. suaveolens* batches of different origin in higher ratios: 22.65% [68] and 13.60% [69]. This differs from our findings and refers to wide, probably genotype-dependent quantitative diversity in the EO composition of this species.

### Soil properties and microbial composition

Despite the similar sandy-loam texture of all soils, their chemical composition differed substantially among *Mentha* species and compared to bulk soil. As shown by the distinct nutrient profiles (Table 1), *M. spicata* B17 soils contained significantly less nitrogen and phosphorus, which may underlie the observed microbial shifts and lower bacterial diversity. Such plant-specific effects on soil chemistry are consistent with earlier findings that aromatic plants alter nutrient availability and pH through differential root exudation and EO secretion, thereby influencing the surrounding microbiota [26].

All three *Mentha* rhizospheres shared a substantial bacterial and fungal core; however, each species enriched distinct taxa through host-mediated selection. The low bacterial richness but high number of unique ASVs observed in *M. spicata* B17 suggests a stronger selective filter, potentially driven by nutrient limitation and antimicrobial components of its EOs. In contrast, the more nutrient-enriched rhizosphere of *M. × villosa* B10 supported a balanced, diverse community, while *M. suaveolens* J17 exhibited intermediate features. Similar host-specific structuring has been reported within the *Lamiaceae*, where *Mentha*, *Thymus*, and *Salvia* maintained distinct rhizosphere assemblages even under identical soil conditions [26]. Such differences have been attributed to the influence of secondary metabolites, particularly EOs, which act as selective agents that stimulate or inhibit specific microbial taxa [14,47].

### Composition and potential functional roles of bacterial and fungal communities

The bacterial communities of *Mentha* rhizospheres were dominated by Actinomycetota, Pseudomonadota, Acidobacteriota, Bacillota, and Chloroflexota, together accounting for more than 80% of all sequences. These phyla are typical of rhizosphere ecosystems and play key roles in nutrient cycling, consistent with findings from other *Lamiaceae* species [26]. Actinomycetota frequently dominate or co-dominate in the rhizospheres of diverse plant species [70–72]. Members of this phylum, such as *Streptomyces*, are prolific producers of antibiotics and extracellular enzymes that contribute to pathogen suppression, organic-matter turnover, and the maintenance of soil health [70,73]. Pseudomonadota represented the second most abundant group, comprising many plant growth-promoting rhizobacteria (PGPR) with abilities such as phosphate solubilization, nitrogen fixation, siderophore production, and pathogen inhibition. Fluorescent *Pseudomonas* strains isolated from *M. piperita* have been reported to stimulate plant growth and modify EO composition via volatile organic compounds [74,75], suggesting a feedback mechanism between rhizosphere microbiota and secondary metabolism. Acidobacteriota, the third most abundant phylum, are ubiquitous chemoorganoheterotrophic bacteria capable of metabolizing a broad range of carbon sources, contributing to organic-matter decomposition and nutrient cycling in soils [76,77]. Comparable taxonomic profiles have been reported for other aromatic plants, such as *Thymus vulgaris* and *Origanum vulgare*, where EO components selectively enriched *Bacillu*s, *Pseudomonas*, and *Streptomyces* involved in detoxification and terpenoid biotransformation [17,47]. Altogether, these shared taxa suggest the existence of a functionally conserved core microbiome associated with the chemically complex rhizosphere of *Lamiaceae* plants.

Fungal assemblages exhibited a similarly structured yet more host-specific pattern compared to bacteria. Across all *Mentha* species, Ascomycota dominated (87.44% on average), followed by Mortierellomycota (7.09%) and Basidiomycota (2.11%). While these phyla were present in all samples, their relative abundances varied markedly among species. The rhizosphere of *M. spicata* B17 contained significantly less Ascomycota but higher proportions of Mortierellomycota, Rozellomycota, and Glomeromycota. This shift toward a more diverse non-Ascomycota assemblage suggests a species-specific restructuring of the fungal community, potentially driven by cultivar-associated differences in root traits, secondary metabolite profiles, and soil nutrient status. Although Glomeromycota accounted for only 0.98% of total sequences, they occurred exclusively in *Mentha* rhizosphere soils, where their abundance followed the trend *M. spicata* > *M. × villosa* > *M. suaveolens*. This pattern mirrored AMF colonization rates observed microscopically and is consistent with earlier reports of strong AMF variability within *Lamiaceae* [78]. Such variation is often linked to differences in root architecture, nutrient availability, and plant chemical phenotype. Recent studies have highlighted the dual role of AMF as biostimulants enhancing plant stress tolerance and as modulators of secondary metabolism, particularly terpenoid biosynthesis, in medicinal and aromatic plants [79–81]. These interactions suggest that *Mentha*-AMF associations not only facilitate nutrient uptake but also may be associated with coordinated changes in plant metabolism and rhizosphere community structure.

### Microbial diversity and species-specific microbiota in *Mentha* rhizospheres

Alpha-diversity indices revealed a clear rhizosphere effect, with greater richness and evenness than in bulk soil. It should be noted that bulk soil samples originated from mechanically maintained inter-row strips and therefore represent a managed baseline rather than undisturbed natural soil. Consequently, the observed rhizosphere effects reflect differences between plant-associated soil and cultivated inter-row soil rather than comparisons with pristine background soil. *M. × villosa* B10 and *M. suaveolens* J17 supported the most diverse microbial consortia, while *M. spicata* B17 again showed the lowest diversity, reflecting its lower nutrient levels and its distinct chemical profile characterized by high L-carvone content, which was associated with reduced diversity metrics. Beta-diversity clustering confirmed host-driven differentiation, with *M. spicata* B17 communities clearly distinct from those of the other two species and from bulk soil. Similar genotype-specific structuring has been observed in *Thymus*, *Ocimum*, and *Salvia*, emphasizing the strong role of secondary metabolites in microbial filtering [17,26].

Differential abundance analyses (DESeq2 and LEfSe) further demonstrated pronounced compositional differentiation between bulk soil and rhizosphere samples. Gram-positive taxa, particularly Bacillota (Bacilli, Bacillales), were enriched in bulk soil, while rhizospheres favored Gram-negative groups such as Pseudomonadota and Acidobacteriota. This pattern is consistent with known physiological differences in tolerance to hydrophobic secondary metabolites, making them more resistant to compounds like carvone or limonene [82–85]. In contrast, Gram-positive spore formers were relatively more abundant in bulk soil, where nutrient limitation rather than plant-associated chemical factors may play a dominant role. Similar Gram-type-driven microbial differentiation has been described in other aromatic and medicinal plants, including *Thymus vulgaris* and *Origanum vulgare*, suggesting that plant chemical profiles are frequently associated with structuring patterns in *Lamiaceae* rhizosphere microbiomes [17,26,47].

When comparing rhizosphere communities among the three *Mentha* species, clear species-specific signatures emerged from both bacterial and fungal datasets. Pairwise DESeq2 comparisons revealed that *M. × villosa* B10 and *M. suaveolens* J17 harbored the most similar bacterial assemblages, differing in only 2.7% of their ASVs, whereas *M. spicata* B17 showed extensive divergence from both, with nearly 40% of ASVs differing significantly. These findings are consistent with α- and β-diversity analyses, confirming that the *M. spicata* B17 rhizosphere harbors the most distinct microbial profile among the examined *Mentha* species.

In *M. suaveolens* J17, the main bacterial biomarkers belonged to the phyla Chloroflexota (Anaerolineae, Caldilineales, *Caldilineaceae*) and Actinomycetota (*Intrasporangiaceae*). Similarly, the *M. × villosa* B10 rhizosphere was also characterized by members of Chloroflexota (uncultured JG30-KF-CM66) and Actinomycetota (including *Desertimonas* and *Microlunatus*), though representing different taxonomic groups than in *M. suaveolens* J17. In contrast, the *M. spicata* B17 rhizosphere displayed the most distinct biomarker profile, dominated by Gram-negative taxa from Myxococcota, Acidobacteriota, and Pseudomonadota (e.g., *Pedomicrobium*, *Rhodoplanes*), along with members of *Steroidobacteraceae*, *Thermoactinomycetaceae*, and *Candidatus Udaeobacter*. Such enrichment of Gram-negative taxa may be associated with the distinctive chemotype of *M. spicata* B17 and its correlated environmental conditions.

Fungal communities showed analogous but more host-specific patterns than bacteria. The LEfSe analysis supported these observations, confirming that *M. × villosa* B10 and *M. suaveolens* J17 were primarily associated with Ascomycota biomarkers, whereas the *M. spicata* B17 rhizosphere displayed a more complex profile that also included biomarkers from Basidiomycota and Glomeromycota. Notably, *Diversispora* (Glomeromycota) was identified as a characteristic biomarker of *M. spicata* B17, in line with the molecular evidence of AMF presence. These concordant results indicate that differential abundance analyses reinforced the previously observed species-specific patterns in fungal community composition.

Taken together, diversity metrics, ordination analyses, and differential abundance results consistently identify *M. spicata* B17 as the most divergent rhizosphere system, whereas *M. × villosa* B10 and *M. suaveolens* J17 display comparatively more similar microbial patterns.

### Associations between soil nutrients, essential-oil chemistry, and rhizosphere microbiota

Correlation and redundancy analyses revealed clear associations between soil physicochemical parameters, EO composition, and the structure of bacterial and fungal communities in the rhizosphere soils of the three *Mentha* taxa. Among the tested soil variables, humus content emerged as the most consistent positive predictor of microbial richness and diversity, confirming that organic carbon availability is a primary driver of rhizosphere microbiome activity [1,86]. Bacterial α-diversity indices (observed species, Chao1, Shannon) correlated positively with humus, indicating that carbon-rich environments sustain broader heterotrophic assemblages, including Actinomycetota and Pseudomonadota. Fungal richness showed a similar response, consistent with the role of organic matter in maintaining saprotrophic and mycorrhizal taxa through enhanced carbon and nutrient supply. Besides humus, phosphorus and potassium significantly contributed to community structuring, while sodium showed a moderate positive correlation with bacterial richness, possibly reflecting mild osmotic stimulation. Conversely, higher pH and Na⁺ were weakly but negatively related to fungal evenness, in agreement with observations from *Lavandula* rhizospheres where elevated pH reduced fungal diversity [25].

Redundancy analysis (RDA) confirmed humus, P, and K as the dominant soil drivers, jointly explaining over 76.2% of bacterial and 53.5% of fungal community variation (p = 0.001). These patterns indicate that nutrient-rich, carbon-enriched microenvironments favor copiotrophic taxa involved in nutrient and organic-matter turnover [87], while fungal assemblages respond similarly but less strongly to these gradients.

Beyond soil chemistry, major EO constituents (L-carvone and limonene) also aligned with microbial community shifts. For bacteria, the constrained RDA model was significant (p = 0.004), with these monoterpenes together explaining 65.5% of total variance (L-carvone: p = 0.009; limonene: p = 0.018). For fungi, the reduced model accounted for 48.3% of variation (L-carvone: p = 0.012; limonene: p = 0.006), revealing a weaker but consistent trend. In ordination space, L-carvone and limonene vectors were directed toward *M. × villosa* B10 and *M. spicata* B17, while *M. suaveolens* J17 separated along the opposite axis – consistent with its different EO content. These results indicate that cultivar-specific EO profiles are statistically associated with community differentiation patterns observed alongside soil nutrient gradients. These associations appeared stronger for bacterial communities, whereas fungal communities showed comparatively tighter correlations with humus and phosphorus gradients.

Altogether, the balance of humus, phosphorus, and potassium, together with species-specific EO profiles, is associated with structuring patterns in the *Mentha* rhizosphere microbiota. Nutrient-driven and metabolite-driven selection pressures interact to form a complex chemical-ecological network that regulates the diversity, stability, and functional potential of these aromatic plant rhizospheres, a pattern consistent with other *Lamiaceae* such as *Lavandula* [25].

Despite the clear compositional patterns observed, certain limitations should be considered. EO composition was characterized from aerial tissues and not directly measured in rhizosphere soil or as root exudates, the primary chemical mediators at the root-soil interface. Accordingly, the reported EO-microbiome relationships should be interpreted as correlations with plant chemotype rather than direct measurements of rhizosphere chemical exposure.

In addition, the study included three biological replicates per cultivar, a replication level typical of field-based microbiome studies but inherently limiting statistical power and effect size precision. Although multiple analytical approaches supported consistent trends, correlations should be interpreted cautiously. Broader spatial or temporal sampling would further strengthen the generality of these findings. Furthermore, as samples were collected during a single growing season and at one phenological stage, the study represents a season-specific snapshot of *Mentha* rhizosphere communities; multi-year and multi-stage sampling would be required to assess the temporal stability of the observed patterns.

## Conclusion

This study demonstrates that even closely related *Mentha* species are associated with distinct rhizosphere microbiota under similar field conditions. Variations in bacterial and fungal diversity, community structure, and AMF colonization were closely associated with both soil nutrient profiles and essential oil chemotypes. Bacterial communities were most strongly correlated with by humus, phosphorus, and potassium availability, whereas fungal diversity exhibited weaker correlations with soil parameters. The dominance of L-carvone- and limonene-rich profiles in *M. × villosa* B10 and *M. spicata* B17, and cis-piperitone-epoxide dominance in *M. suaveolens* J17, corresponded to distinct microbial assemblages, indicating genotype-associated chemical differentiation patterns. Overall, our findings highlight that even subtle genetic and metabolic differences among aromatic plants can be associated with substantial variation in rhizosphere microbial composition, offering new avenues for targeted microbiome management in medicinal and aromatic crop systems.

## Supporting information

S1 Fig Summary of differentially abundant ASVs between bulk soil and *Mentha* rhizosphere soils identified by DESeq2. A: bacterial ASVs; B: fungal ASVs. Each pie chart shows the proportion of ASVs significantly enriched in bulk soil or in rhizosphere soils (padj < 0.05), or not significantly different between groups. Differential abundance analysis was conducted using three biological replicates per cultivar (total n = 9 rhizosphere samples) and three bulk soil samples (n = 3).
(TIF)

S2 Fig Summary of differentially abundant ASVs among the rhizosphere soils of the three *Mentha* cultivars, identified by DESeq2 (n = 3 biological replicates per cultivar). Pairwise comparisons of bacterial (upper panel) and fungal (lower panel) ASVs between B10 and J17, B10 and B17, and B17 and J17 are shown. Each stacked bar represents the proportion of ASVs significantly enriched in either cultivar (padj < 0.05) or not significantly different between cultivars.
(TIF)

S1 Table Comparison of Hungarian standard (MSZ) soil analysis methods and their international equivalents. (TIF)

S2 Table The basic sequence data of bacterial communities found in all samples. (XLSX)

S3 Table The basic sequence data of fungal communities found in all samples. (XLSX)

## References

[1] Philippot L, Raaijmakers JM, Lemanceau P, van der Putten WH. Going back to the roots: the microbial ecology of the rhizosphere. Nat Rev Microbiol. 2013;11(11):789–99. doi: 10.1038/nrmicro3109 24056930

[2] Berendsen RL, Pieterse CMJ, Bakker PAHM. The rhizosphere microbiome and plant health. Trends Plant Sci. 2012;17(8):478–86. doi: 10.1016/j.tplants.2012.04.001 22564542

[3] Beneduzi A, Ambrosini A, Passaglia LMP. Plant growth-promoting rhizobacteria (PGPR): Their potential as antagonists and biocontrol agents. Genet Mol Biol. 2012;35(4 (suppl)):1044–51. doi: 10.1590/s1415-47572012000600020 23411488 PMC3571425

[4] Mendes R, Garbeva P, Raaijmakers JM. The rhizosphere microbiome: significance of plant beneficial, plant pathogenic, and human pathogenic microorganisms. FEMS Microbiol Rev. 2013;37(5):634–63. doi: 10.1111/1574-6976.12028 23790204

[5] Bonfante P, Anca I-A. Plants, mycorrhizal fungi, and bacteria: a network of interactions. Annu Rev Microbiol. 2009;63:363–83. doi: 10.1146/annurev.micro.091208.073504 19514845

[6] Lian W-H, Mohamad OAA, Dong L, Zhang L-Y, Wang D, Liu L, et al. Culturomics- and metagenomics-based insights into the microbial community and function of rhizosphere soils in Sinai desert farming systems. Environ Microbiome. 2023;18(1):4. doi: 10.1186/s40793-023-00463-3 36639807 PMC9840269

[7] Chaparro JM, Badri DV, Vivanco JM. Rhizosphere microbiome assemblage is affected by plant development. ISME J. 2014;8(4):790–803. doi: 10.1038/ismej.2013.196 24196324 PMC3960538

[8] Sasse J, Martinoia E, Northen T. Feed your friends: do plant exudates shape the root microbiome? Trends in plant science. 2018;23(1):25–41. 29050989 10.1016/j.tplants.2017.09.003

[9] Smith SE, Read DJ. Mycorrhizal symbiosis: Academic press; 2010.

[10] Brundrett M. Diversity and classification of mycorrhizal associations. Biol Rev Camb Philos Soc. 2004;79(3):473–95. doi: 10.1017/s1464793103006316 15366760

[11] Li L, Huang J, Liu Y, Zhang Q, Han Q, Liu Y, et al. Analysis of microbial community composition and diversity in the rhizosphere of Salvia miltiorrhiza at different growth stages. Int Microbiol. 2025;28(2):277–88. doi: 10.1007/s10123-024-00542-6 38833100

[12] Knight R, Vrbanac A, Taylor BC, Aksenov A, Callewaert C, Debelius J, et al. Best practices for analysing microbiomes. Nat Rev Microbiol. 2018;16(7):410–22. doi: 10.1038/s41579-018-0029-9 29795328

[13] Gilbert JA, Jansson JK, Knight R. The Earth Microbiome project: successes and aspirations. BMC Biol. 2014;12:69. doi: 10.1186/s12915-014-0069-1 25184604 PMC4141107

[14] Bakkali F, Averbeck S, Averbeck D, Idaomar M. Biological effects of essential oils--a review. Food Chem Toxicol. 2008;46(2):446–75. doi: 10.1016/j.fct.2007.09.106 17996351

[15] Kuzyakov Y, Razavi BS. Rhizosphere size and shape: Temporal dynamics and spatial stationarity. Soil Biology and Biochemistry. 2019;135:343–60. doi: 10.1016/j.soilbio.2019.05.011

[16] Vaou N, Stavropoulou E, Voidarou C, Tsigalou C, Bezirtzoglou E. Towards Advances in Medicinal Plant Antimicrobial Activity: A Review Study on Challenges and Future Perspectives. Microorganisms. 2021;9(10):2041. doi: 10.3390/microorganisms9102041 34683362 PMC8541629

[17] Misra P, Maji D, Awasthi A, Pandey SS, Yadav A, Pandey A, et al. Vulnerability of Soil Microbiome to Monocropping of Medicinal and Aromatic Plants and Its Restoration Through Intercropping and Organic Amendments. Front Microbiol. 2019;10:2604. doi: 10.3389/fmicb.2019.02604 31803153 PMC6877478

[18] Tafrihi M, Imran M, Tufail T, Gondal TA, Caruso G, Sharma S, et al. The Wonderful Activities of the Genus Mentha: Not Only Antioxidant Properties. Molecules. 2021;26(4):1118. doi: 10.3390/molecules26041118 33672486 PMC7923432

[19] Tavaszi-Sárosi S, Sfaxi A, Juhász Á, Radácsi P, Patonay K. Chemical and biological properties of Mentha × villosa Huds. (mojito mint) and its parental species—Mentha spicata L. (spearmint) and Mentha suaveloens Ehrh. (apple mint)—a review. Phytochem Rev. 2025;25(3):2065–116. doi: 10.1007/s11101-025-10136-3

[20] Sfaxi A, Tavaszi-Sárosi S, Flórián K, Patonay K, Radácsi P, Juhász Á. Comparative Evaluation of Different Mint Species Based on Their In Vitro Antioxidant and Antibacterial Effect. Plants (Basel). 2025;14(1):105. doi: 10.3390/plants14010105 39795364 PMC11723094

[21] Kowalczyk A, Piątkowska E, Kuś P, Marijanović Z, Jerković I, Tuberoso CIG, et al. Volatile compounds and antibacterial effect of commercial mint cultivars - chemotypes and safety. Industrial Crops and Products. 2021;166:113430. doi: 10.1016/j.indcrop.2021.113430

[22] Raveau R, Fontaine J, Lounès-Hadj Sahraoui A. Essential Oils as Potential Alternative Biocontrol Products against Plant Pathogens and Weeds: A Review. Foods. 2020;9(3):365. doi: 10.3390/foods9030365 32245234 PMC7143296

[23] Ainalidou A, Bouzoukla F, Menkissoglu-Spiroudi U, Vokou D, Karamanoli K. Impacts of Decaying Aromatic Plants on the Soil Microbial Community and on Tomato Seedling Growth and Metabolism: Suppression or Stimulation? Plants (Basel). 2021;10(9):1848. doi: 10.3390/plants10091848 34579381 PMC8471824

[24] Checcucci A, Maida I, Bacci G, Ninno C, Bilia AR, Biffi S, et al. Is the plant-associated microbiota of Thymus spp. adapted to plant essential oil? Research in Microbiology. 2017;168(3):276–82. 27884782 10.1016/j.resmic.2016.11.004

[25] Deng X, Shi R, Elnour RO, Guo Z, Wang J, Liu W, et al. Analysis of rhizosphere fungal diversity in lavender at different planting years based on high-throughput sequencing technology. PLoS One. 2024;19(10):e0310929. doi: 10.1371/journal.pone.0310929 39361671 PMC11449376

[26] Zharkova EK, Vankova AA, Selitskaya OV, Malankina EL, Drenova NV, Zhelezova AD, et al. Bacterial Communities of Lamiacea L. Medicinal Plants: Structural Features and Rhizosphere Effect. Microorganisms. 2023;11(1):197. doi: 10.3390/microorganisms11010197 36677489 PMC9865931

[27] Çolak Esetlili B, Esetlili MT, Emir K, Eröz M. Sustainable Agrivoltaic Farming: The Role of Mycorrhiza in Promoting Mint Cultivation and High-Quality Essential Oil Production. Sustainability. 2025;17(12):5516. doi: 10.3390/su17125516

[28] Heydarizadeh P, Zahedi M, Sabzalian MR, Ataii E. Mycorrhizal infection, essential oil content and morpho-phenological characteristics variability in three mint species. Scientia Horticulturae. 2013;153:136–42. doi: 10.1016/j.scienta.2013.01.014

[29] Urcoviche RC, Gazim ZC, Dragunski DC, Barcellos FG, Alberton O. Plant growth and essential oil content of Mentha crispa inoculated with arbuscular mycorrhizal fungi under different levels of phosphorus. Industrial Crops and Products. 2015;67:103–7. doi: 10.1016/j.indcrop.2015.01.016

[30] Karagiannidis N, Thomidis T, Lazari D, Panou-Filotheou E, Karagiannidou C. Effect of three Greek arbuscular mycorrhizal fungi in improving the growth, nutrient concentration, and production of essential oils of oregano and mint plants. Scientia Horticulturae. 2011;129(2):329–34. doi: 10.1016/j.scienta.2011.03.043

[31] Vandendool H, Kratz PD. A Generalization of the retention index system including linear temperature programmed gas-liquid partition chromatography. J Chromatogr. 1963;11:463–71. doi: 10.1016/s0021-9673(01)80947-x 14062605

[32] Vierheilig H, Coughlan A, Wyss U, Piche Y. Ink and vinegar, a simple staining technique for arbuscular-mycorrhizal fungi. Appl Environ Microbiol. 1998;64(12):5004–7. doi: 10.1128/AEM.64.12.5004-5007.1998 9835596 PMC90956

[33] Giovannetti M, Mosse B. An evaluation of techniques for measuring vesicular arbuscular mycorrhizal infection in roots. New phytologist. 1980:489–500.

[34] Klindworth A, Pruesse E, Schweer T, Peplies J, Quast C, Horn M, et al. Evaluation of general 16S ribosomal RNA gene PCR primers for classical and next-generation sequencing-based diversity studies. Nucleic Acids Res. 2013;41(1):e1. doi: 10.1093/nar/gks808 22933715 PMC3592464

[35] Illumina. Fungal Metagenomic Sequencing Demonstrated Protocol.

[36] Illumina. 16S Metagenomic Sequencing Library Preparation. 2013.

[37] Escudié F, Auer L, Bernard M, Mariadassou M, Cauquil L, Vidal K, et al. FROGS: Find, Rapidly, OTUs with Galaxy Solution. Bioinformatics. 2018;34(8):1287–94. doi: 10.1093/bioinformatics/btx791 29228191

[38] Rognes T, Flouri T, Nichols B, Quince C, Mahé F. VSEARCH: a versatile open source tool for metagenomics. PeerJ. 2016;4:e2584. doi: 10.7717/peerj.2584 27781170 PMC5075697

[39] Mahé F, Rognes T, Quince C, de Vargas C, Dunthorn M. Swarm: robust and fast clustering method for amplicon-based studies. PeerJ. 2014;2:e593. doi: 10.7717/peerj.593 25276506 PMC4178461

[40] McGinnis S, Madden TL. BLAST: at the core of a powerful and diverse set of sequence analysis tools. Nucleic Acids Res. 2004;32(Web Server issue):W20–5. doi: 10.1093/nar/gkh435 15215342 PMC441573

[41] Quast C, Pruesse E, Yilmaz P, Gerken J, Schweer T, Yarza P, et al. The SILVA ribosomal RNA gene database project: improved data processing and web-based tools. Nucleic Acids Res. 2013;41(Database issue):D590–6. doi: 10.1093/nar/gks1219 23193283 PMC3531112

[42] Abarenkov K, Nilsson RH, Larsson K-H, Taylor AF, May TW, Frøslev TG, et al. The UNITE database for molecular identification and taxonomic communication of fungi and other eukaryotes: sequences, taxa and classifications reconsidered. Nucleic Acids Research. 2024;52(D1):D791–D7. 10.1093/nar/gkad1039 PMC10767974 37953409

[43] McMurdie PJ, Holmes S. phyloseq: an R package for reproducible interactive analysis and graphics of microbiome census data. PLoS One. 2013;8(4):e61217. doi: 10.1371/journal.pone.0061217 23630581 PMC3632530

[44] Love MI, Huber W, Anders S. Moderated estimation of fold change and dispersion for RNA-seq data with DESeq2. Genome Biol. 2014;15(12):550. doi: 10.1186/s13059-014-0550-8 25516281 PMC4302049

[45] Segata N, Izard J, Waldron L, Gevers D, Miropolsky L, Garrett WS, et al. Metagenomic biomarker discovery and explanation. Genome Biol. 2011;12(6):R60. doi: 10.1186/gb-2011-12-6-r60 21702898 PMC3218848

[46] Fox J, Leanage A. R and the Journal of Statistical Software. J Stat Soft. 2016;73(2). doi: 10.18637/jss.v073.i02

[47] Pang Z, Chen J, Wang T, Gao C, Li Z, Guo L, et al. Linking plant secondary metabolites and plant microbiomes: a review. Frontiers in plant science. 2021;12:621276. 33737943 10.3389/fpls.2021.621276 PMC7961088

[48] Ghotbi-Ravandi AA, Shariatmadari Z, Riahi H, Hassani SB, Heidari F, Ghorbani Nohooji M. Enhancement of Essential Oil Production and Expression of Some Menthol Biosynthesis-Related Genes in Mentha piperita Using Cyanobacteria. Iran J Biotechnol. 2023;21(4):e3550. doi: 10.30498/ijb.2023.368377.3550 38269195 PMC10804067

[49] Jamwal VL, Rather IA, Ahmed S, Kumar A, Gandhi SG. Changing Rhizosphere Microbial Community and Metabolites with Developmental Stages of Coleus barbatus. Microorganisms. 2023;11(3):705. doi: 10.3390/microorganisms11030705 36985280 PMC10056624

[50] Steinberger Y, Doniger T, Sherman C, Jeyaraman M, Applebaum I. Soil Bacterial Community of Medicinal Plant Rhizosphere in a Mediterranean System. Agriculture. 2024;14(5):664. doi: 10.3390/agriculture14050664

[51] Cappellari LDR, Santoro MV, Schmidt A, Gershenzon J, Banchio E. Induction of essential oil production in Mentha x piperita by plant growth promoting bacteria was correlated with an increase in jasmonate and salicylate levels and a higher density of glandular trichomes. Plant Physiol Biochem. 2019;141:142–53. doi: 10.1016/j.plaphy.2019.05.030 31163341

[52] Hayes J, Stavanja M, Lawrence B. Mint. The Genus Mentha. Boca Raton, CRC Press Taylor and Francis; 2007.

[53] Baser KHC, Kürkçüoglu M, Tarimcilar G, Kaynak G. Essential Oils ofMenthaSpecies from Northern Turkey. Journal of Essential Oil Research. 1999;11(5):579–88. doi: 10.1080/10412905.1999.9701218

[54] Rasooli I, Gachkar L, Yadegarinia D, Bagher Rezaei M, Alipoor Astaneh S. Antibacterial and antioxidative characterisation of essential oils from Mentha piperita and Mentha spicata grown in Iran. Acta alimentaria. 2008;37(1):41–52.

[55] Chowdhury JU, Nandi NC, Uddin M, Rahman M. Chemical constituents of essential oils from two types of spearmint (Mentha spicata L. and M. cardiaca L.) introduced in Bangladesh. Bangladesh Journal of Scientific and Industrial Research. 2007;42(1):79–82.

[56] Znini M, Bouklah M, Majidi L, Kharchouf S, Aouniti A, Bouyanzer A, et al. Chemical Composition and Inhibitory Effect of Mentha Spicata Essential Oil on the Corrosion of Steel in Molar Hydrochloric Acid. International Journal of Electrochemical Science. 2011;6(3):691–704. doi: 10.1016/s1452-3981(23)15027-9

[57] Fitsiou E, Mitropoulou G, Spyridopoulou K, Tiptiri-Kourpeti A, Vamvakias M, Bardouki H, et al. Phytochemical Profile and Evaluation of the Biological Activities of Essential Oils Derived from the Greek Aromatic Plant Species Ocimum basilicum, Mentha spicata, Pimpinella anisum and Fortunella margarita. Molecules. 2016;21(8):1069. doi: 10.3390/molecules21081069 27537869 PMC6274325

[58] Boukhebti H, Chaker AN, Belhadj H, Sahli F, Ramdhani M, Laouer H, et al. Chemical composition and antibacterial activity of Mentha pulegium L. and Mentha spicata L. essential oils. Der Pharmacia Lettre. 2011;3(4):267–75.

[59] de Sousa Barros A, de Morais SM, Ferreira PAT, Vieira ÍGP, Craveiro AA, dos Santos Fontenelle RO, et al. Chemical composition and functional properties of essential oils from Mentha species. Industrial Crops and Products. 2015;76:557–64.

[60] González Martínez CA. Principais componentes do óleo essencial de acessos de Mentha spp em Brasília e estudo da propagação vegetativa. 2016.

[61] Teles S, Pereira JA, Santos CHB, Menezes RV, Malheiro R, Lucchese AM, et al. Effect of geographical origin on the essential oil content and composition of fresh and dried Mentha×villosa Hudson leaves. Industrial Crops and Products. 2013;46:1–7. doi: 10.1016/j.indcrop.2012.12.009

[62] Lima TC, da Silva TKM, Silva FL, Barbosa-Filho JM, Marques MOM, Santos RLC, et al. Larvicidal activity of Mentha x villosa Hudson essential oil, rotundifolone and derivatives. Chemosphere. 2014;104:37–43. doi: 10.1016/j.chemosphere.2013.10.035 24275151

[63] Vining KJ, Pandelova I, Hummer K, Bassil N, Contreras R, Neill K, et al. Genetic diversity survey of Mentha aquatica L. and Mentha suaveolens Ehrh., mint crop ancestors. Genet Resour Crop Evol. 2019;66(4):825–45. doi: 10.1007/s10722-019-00750-4

[64] Zekri N, Elazzouzi H, Ailli A, Gouruch AA, Radi FZ, El Belghiti MA, et al. Physicochemical Characterization and Antioxidant Properties of Essential Oils of M. pulegium (L.), M. suaveolens (Ehrh.) and M. spicata (L.) from Moroccan Middle-Atlas. Foods. 2023;12(4):760. doi: 10.3390/foods12040760 36832835 PMC9955515

[65] Oumzil H, Ghoulami S, Rhajaoui M, Ilidrissi A, Fkih-Tetouani S, Faid M, et al. Antibacterial and antifungal activity of essential oils of Mentha suaveolens. Phytother Res. 2002;16(8):727–31. doi: 10.1002/ptr.1045 12458474

[66] Poli J-P, Guinoiseau E, de Rocca Serra D, Sutour S, Paoli M, Tomi F, et al. Anti-Quorum Sensing Activity of 12 Essential Oils on chromobacterium violaceum and Specific Action of cis-cis-p-Menthenolide from Corsican Mentha suaveolens ssp. Insularis. Molecules. 2018;23(9):2125. doi: 10.3390/molecules23092125 30142938 PMC6225197

[67] Maffei M, Bertea C, Mucciarelli M. Anatomy, physiology, biosynthesis, molecular biology, tissue culture, and biotechnology of mint essential oil production. Mint: the genus Mentha. 2007:41–85.

[68] Elansary HO, Ashmawy NA. Essential Oils of Mint between Benefits and Hazards. Journal of Essential Oil Bearing Plants. 2013;16(4):429–38. doi: 10.1080/0972060x.2013.813279

[69] Benali T, Bouyahya A, Habbadi K, Zengin G, Khabbach A, Achbani EH, et al. Chemical composition and antibacterial activity of the essential oil and extracts of Cistus ladaniferus subsp. ladanifer and Mentha suaveolens against phytopathogenic bacteria and their ecofriendly management of phytopathogenic bacteria. Biocatalysis and Agricultural Biotechnology. 2020;28:101696. doi: 10.1016/j.bcab.2020.101696

[70] Boukhatem ZF, Merabet C, Tsaki H. Plant growth promoting actinobacteria, the most promising candidates as bioinoculants? Frontiers in Agronomy. 2022;4:849911.

[71] Oberhofer M, Hess J, Leutgeb M, Gössnitzer F, Rattei T, Wawrosch C, et al. Exploring Actinobacteria Associated With Rhizosphere and Endosphere of the Native Alpine Medicinal Plant Leontopodium nivale Subspecies alpinum. Front Microbiol. 2019;10:2531. doi: 10.3389/fmicb.2019.02531 31781058 PMC6857621

[72] Chen J, Li N, Chang J, Ren K, Zhou J, Yang G. Taxonomic Structure of Rhizosphere Bacterial Communities and Its Association With the Accumulation of Alkaloidal Metabolites in Sophora flavescens. Front Microbiol. 2021;12:781316. doi: 10.3389/fmicb.2021.781316 34970241 PMC8712762

[73] Narsing Rao MP, Lohmaneeratana K, Bunyoo C, Thamchaipenet A. Actinobacteria-Plant Interactions in Alleviating Abiotic Stress. Plants (Basel). 2022;11(21):2976. doi: 10.3390/plants11212976 36365429 PMC9658302

[74] Santoro M, Cappellari LdR, Giordano W, Banchio E. Plant growth‐promoting effects of native Pseudomonas strains on Mentha piperita (peppermint): an in vitro study. Plant Biology. 2015;17(6):1218–26. 26012535 10.1111/plb.12351

[75] Santoro MV, Bogino PC, Nocelli N, Cappellari LDR, Giordano WF, Banchio E. Analysis of Plant Growth-Promoting Effects of Fluorescent Pseudomonas Strains Isolated from Mentha piperita Rhizosphere and Effects of Their Volatile Organic Compounds on Essential Oil Composition. Front Microbiol. 2016;7:1085. doi: 10.3389/fmicb.2016.01085 27486441 PMC4949228

[76] Kielak AM, Barreto CC, Kowalchuk GA, van Veen JA, Kuramae EE. The Ecology of Acidobacteria: Moving beyond Genes and Genomes. Front Microbiol. 2016;7:744. doi: 10.3389/fmicb.2016.00744 27303369 PMC4885859

[77] Zhang Y, Cong J, Lu H, Li G, Qu Y, Su X, et al. Community structure and elevational diversity patterns of soil Acidobacteria. J Environ Sci (China). 2014;26(8):1717–24. doi: 10.1016/j.jes.2014.06.012 25108728

[78] Sharma K, Singh M, Srivastava DK, Singh PK. Exploring the Diversity, Root Colonization, and Morphology of Arbuscular Mycorrhizal Fungi in Lamiaceae. J Basic Microbiol. 2025;65(1):e2400379. doi: 10.1002/jobm.202400379 39428672

[79] Delaeter M, Magnin-Robert M, Randoux B, Lounès-Hadj Sahraoui A. Arbuscular Mycorrhizal Fungi as Biostimulant and Biocontrol Agents: A Review. Microorganisms. 2024;12(7):1281. doi: 10.3390/microorganisms12071281 39065050 PMC11278648

[80] Ahmad N, Malik MA, Bhat MY, Wani AH. Ecological assessment of arbuscular mycorrhizal fungi in medicinal plants across different seasons and locations in Kashmir Valley, India. Environmental Challenges. 2025;20:101224. doi: 10.1016/j.envc.2025.101224

[81] Israel A, Langrand J, Fontaine J, Lounès-Hadj Sahraoui A. Significance of Arbuscular Mycorrhizal Fungi in Mitigating Abiotic Environmental Stress in Medicinal and Aromatic Plants: A Review. Foods. 2022;11(17):2591. doi: 10.3390/foods11172591 36076777 PMC9455813

[82] Burt S. Essential oils: their antibacterial properties and potential applications in foods--a review. Int J Food Microbiol. 2004;94(3):223–53. doi: 10.1016/j.ijfoodmicro.2004.03.022 15246235

[83] Nazzaro F, Fratianni F, De Martino L, Coppola R, De Feo V. Effect of essential oils on pathogenic bacteria. Pharmaceuticals (Basel). 2013;6(12):1451–74. doi: 10.3390/ph6121451 24287491 PMC3873673

[84] Angane M, Swift S, Huang K, Butts CA, Quek SY. Essential Oils and Their Major Components: An Updated Review on Antimicrobial Activities, Mechanism of Action and Their Potential Application in the Food Industry. Foods. 2022;11(3):464. doi: 10.3390/foods11030464 35159614 PMC8833992

[85] Aelenei P, Miron A, Trifan A, Bujor A, Gille E, Aprotosoaie AC. Essential Oils and Their Components as Modulators of Antibiotic Activity against Gram-Negative Bacteria. Medicines (Basel). 2016;3(3):19. doi: 10.3390/medicines3030019 28930130 PMC5456245

[86] Fierer N. Embracing the unknown: disentangling the complexities of the soil microbiome. Nat Rev Microbiol. 2017;15(10):579–90. doi: 10.1038/nrmicro.2017.87 28824177

[87] Zheng Q, Hu Y, Zhang S, Noll L, Böckle T, Dietrich M, et al. Soil multifunctionality is affected by the soil environment and by microbial community composition and diversity. Soil Biol Biochem. 2019;136:107521. doi: 10.1016/j.soilbio.2019.107521 31700196 PMC6837881

