# Supplementary figures and images for: Microbial communities in the rhizosphere of three Mentha species: Links to soil properties and essential oil profiles
Source: PLoS One. 2026 Jul 31;21(7):e0354132. doi: 10.1371/journal.pone.0354132 (PMC13426939; doi:10.1371/journal.pone.0354132)

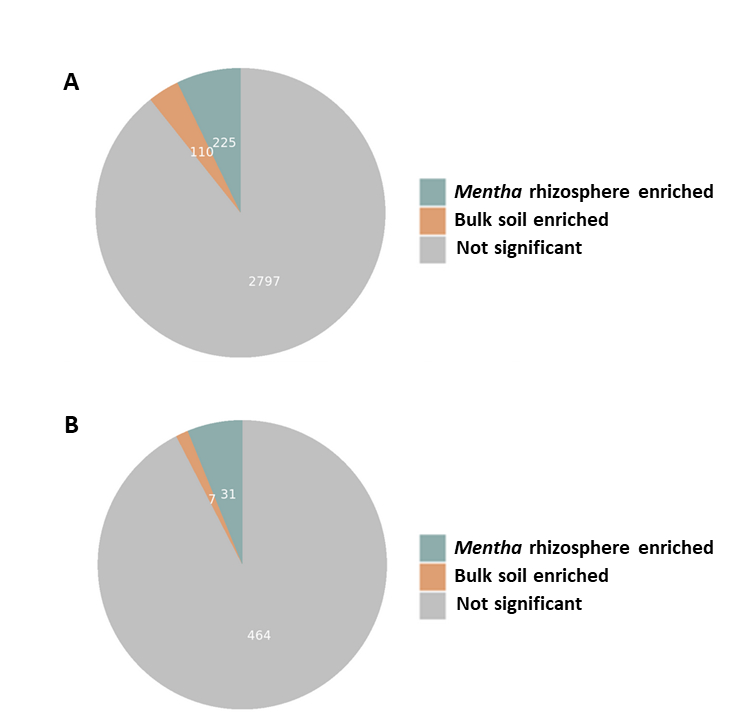

Supplement: S1 Fig — A: bacterial ASVs; B: fungal ASVs. Each pie chart shows the proportion of ASVs significantly enriched in bulk soil or in rhizosphere soils (padj < 0.05), or not significantly different between groups. Differential abundance analysis was conducted using three biological replicates per cultivar (total n = 9 rhizosphere samples) and three bulk soil samples (n = 3). (TIF) [file pone.0354132.s001.tif]

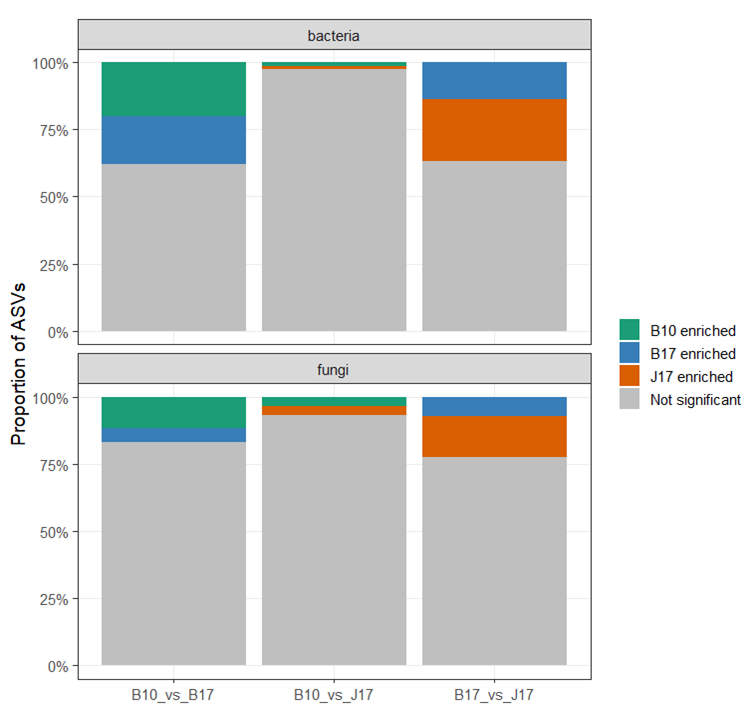

Supplement: S2 Fig — Pairwise comparisons of bacterial (upper panel) and fungal (lower panel) ASVs between B10 and J17, B10 and B17, and B17 and J17 are shown. Each stacked bar represents the proportion of ASVs significantly enriched in either cultivar (padj < 0.05) or not significantly different between cultivars. (TIF) [file pone.0354132.s002.tif]
