# Supplementary material for: Microbial communities in the rhizosphere of three Mentha species: Links to soil properties and essential oil profiles
Source: PLoS One. 2026 Jul 31;21(7):e0354132. doi: 10.1371/journal.pone.0354132 (PMC13426939; doi:10.1371/journal.pone.0354132)
Supplement: Attachment — Submitted filename: Reviewer Report - PlosOne.pdf [file pone.0354132.s006.pdf]

**Reviewer Report**

**Summary assessment**

This manuscript presents a well-executed field study examining rhizosphere bacterial and fungal communities associated with three closely related *Mentha* taxa cultivated under uniform conditions. The integration of amplicon sequencing (16S rRNA and ITS), soil physicochemical analyses, essential oil profiling, and arbuscular mycorrhizal assessments provides a comprehensive and methodologically sound dataset.

The experimental design is appropriate, the methods are clearly described and reproducible, and the statistical analyses are suitable for the data. The results support the main conclusions. In line with **PLOS ONE**'s focus on technical soundness rather than novelty, this study meets the journal's publication criteria.

**Recommendation:** Minor revision.

**Major comments:**

1. **Essential oil measurements as proxies for rhizosphere chemistry**
   Essential oils were extracted from dried aerial tissues and correlated with rhizosphere microbial communities, whereas root exudates – the primary chemical mediators at the root–soil interface – were not measured. This represents an indirect proxy.
   *Recommendation:* Reframe interpretations throughout the Results and Discussion as **correlational associations with plant chemotype**, rather than direct mechanistic effects. Explicitly acknowledge this limitation in the Methods and Discussion.
2. **Statistical power and sample size**
   The study employs $n = 3$ biological replicates per cultivar, resulting in 12 samples for correlation and multivariate analyses. While common in field microbiome studies, this limits statistical power and the precision of effect estimates.
   *Recommendation:* Explicitly acknowledge this limitation in the Discussion and note that correlations should be interpreted cautiously given sample size. Reporting effect sizes alongside p-values where feasible would improve interpretability.
3. **Multicollinearity in RDA models**
   High variance inflation factors indicate substantial multicollinearity among predictors retained in the RDA models.
   *Recommendation:* Clarify that the RDA results reflect **combined environmental gradients** rather than independent effects of individual variables and briefly justify retaining correlated predictors for descriptive purposes.
4. **Definition of bulk soil**
   "Bulk soil" samples were collected from vegetation-free inter-row strips maintained by mechanical weeding or shallow tillage. This represents a disturbed baseline rather than undisturbed bulk soil.
   *Recommendation:* Clarify sampling distance from plant rows and explicitly acknowledge the disturbed nature of these samples, with brief discussion of implications for interpreting rhizosphere effects.

**Minor comments:**

- Clarify the rarefaction depth used for sequencing normalization and whether any samples were excluded due to low read counts.
- State the rarefaction depth when reporting ASV counts.
- Add sample size ($n$ = 3) to relevant figure captions for transparency.
- For RDA figures, include the percentage of variance explained by each axis and note multicollinearity in the captions.
- Deposit raw sequencing data in a public repository (e.g., NCBI SRA or ENA) and provide accession numbers.

**Final recommendation**

This is solid, well-executed research that makes a valuable contribution to understanding plant-microbe interactions in aromatic medicinal plants. The requested revisions are primarily clarifications and reframings rather than additional experiments or analyses. With these minor revisions, the manuscript will be suitable for publication in **PLOS ONE**.
