# Supplementary material for: Microbial communities in the rhizosphere of three Mentha species: Links to soil properties and essential oil profiles
Source: PLoS One. 2026 Jul 31;21(7):e0354132. doi: 10.1371/journal.pone.0354132 (PMC13426939; doi:10.1371/journal.pone.0354132)
Supplement: Attachment — Submitted filename: Response to Reviewers.docx [file pone.0354132.s009.docx]

**Response to reviewers**:

We thank the Editor and Reviewers for their constructive and insightful comments. We have carefully revised the manuscript accordingly, and all changes are highlighted in the tracked version. Below, we provide a point-by-point response.

**Reviewer 1:**

***1. Essential oil measurements as proxies for rhizosphere chemistry.***

Response: We thank the reviewer for this important and constructive comment. We agree that essential oil (EO) composition determined from aerial plant tissues represents an indirect proxy of rhizosphere chemical conditions and does not directly reflect root exudates, which are the primary mediators at the root-soil interface. In response, we have systematically revised the manuscript to clarify the correlative nature of the EO-microbiome relationships:

*Methods section:*

We have explicitly stated that EO composition was characterized from aerial tissues and not directly measured in rhizosphere soil or as root exudates. The text now clarifies that associations between EO profiles and microbial communities should be interpreted as correlations with plant chemotype rather than direct measurements of rhizosphere chemical drivers. See lines 183-186

*Results section:*

The subsection addressing EO-microbiome relationships has been revised to avoid mechanistic phrasing. Expressions implying direct influence (e.g., “drivers,” “shaping effects”) have been replaced with terminology reflecting statistical association (e.g., “associated with,” “aligned with,” “correlated with”).

*Discussion section:*

Interpretations of EO-related findings have been reframed to emphasize chemotype-associated patterns rather than causal filtering effects. Additionally, a dedicated limitation paragraph has been added at the end of the Discussion explicitly acknowledging that EO measurements represent an indirect proxy and that direct rhizosphere metabolite profiling would be required for mechanistic inference. See lines 901-905

*Abstract and Conclusion:*

Mechanistic wording has been adjusted to ensure consistent correlative framing throughout the manuscript.

We believe these revisions adequately address the reviewer’s concern and clearly position EO-microbiome relationships as correlative associations with plant chemotype rather than direct evidence of rhizosphere chemical causation.

***2. Statistical power and sample size.***

Response: We thank the reviewer for highlighting this important point. We acknowledge that the study design (n = 3 biological replicates per cultivar; 12 samples in total) inherently limits statistical power and the precision of effect size estimation.

In response, we have explicitly addressed this limitation in the Discussion by noting that correlations should be interpreted with appropriate caution given the sample size. See lines 906-910. We also clarified that, although multiple analytical approaches (diversity metrics, differential abundance analyses, and multivariate models) yielded consistent trends, expanded spatial or temporal replication would further strengthen generalizability.

***3. Multicollinearity in RDA models.***

Response: We thank the reviewer for this valuable observation. We acknowledge that variance inflation factors indicated multicollinearity among some predictors retained in the RDA models. In the revised manuscript, we have clarified that the RDA results are interpreted as reflecting combined environmental gradients rather than independent effects of individual variables. Correlated predictors were retained to capture the overall nutrient and chemical context of each cultivar for descriptive purposes. We believe this clarification resolves the concern while preserving the ecological interpretation of the multivariate patterns. See lines 670-672.

***4. Definition of bulk soil***

Response: We thank the reviewer for this important clarification. We have revised the Methods section to specify the sampling distance from plant rows and to explicitly state that bulk soil samples were collected from mechanically maintained inter-row strips. We now clarify that these samples represent managed, disturbed soil rather than undisturbed natural bulk soil. See lines 142-146

In addition, we have briefly addressed this point in the Discussion, noting that the observed rhizosphere effects should be interpreted as contrasts between plant-associated soil and cultivated inter-row soil. We believe this clarification improves transparency and interpretation of the rhizosphere comparisons. See lines 812-816

**Minor comments:**

***Clarify the rarefaction depth used for sequencing normalization and whether any samples were excluded due to low read counts.***

Response: The rarefaction depth used for sequencing normalization has now been explicitly stated in the Methods section. Bacterial 16S datasets were rarefied to 95,742 reads per sample, and fungal ITS datasets to 51,602 reads per sample. No samples were excluded due to low read counts. (See Methods, lines 245–247.)

***State the rarefaction depth when reporting ASV counts.***

Response: We have clarified this point in the revised manuscript. The total ASV counts and Venn analyses reported in the “Base sequence data information” section are based on the full quality-filtered dataset. In contrast, alpha- and beta-diversity metrics were calculated after rarefaction to 95,742 reads per sample (16S) and 51,602 reads per sample (ITS), as described in the (updated) Methods. This distinction has now been explicitly stated in the Results section for clarity. (See Results, lines 322–325.)

***Add sample size (n = 3) to relevant figure captions for transparency.***

Response: Sample size information has been added to all relevant main and supplementary figure captions (Figs. 3–8, Figs. S1–S2) for transparency. In addition, sample size (n = 3 per soil type or cultivar) has been specified in the captions of Tables 1 and 2 where mean ± SD values are reported.

***For RDA figures, include the percentage of variance explained by each axis and note multicollinearity in the captions.***

Response: The percentage of variance explained by RDA1 and RDA2 has been added to the captions of Figures 7 and 8. In addition, multicollinearity among predictors and its interpretation as combined environmental or chemotype-associated gradients are now explicitly noted in the captions. Corresponding numerical values in the Results and Discussion sections have been verified and corrected where necessary to ensure consistency with the RDA plots.

***Deposit raw sequencing data in a public repository (e.g., NCBI SRA or ENA) and provide accession numbers.***

Response: The raw sequencing data have been deposited in the NCBI Sequence Read Archive (SRA) under BioProject accession number PRJNA1418655. The Data Availability Statement has been updated accordingly.

**Reviewer 2:**

***1. Explicitly state what is unknown about Mentha-rhizosphere interactions before introducing your study.***

Response: We appreciate this constructive suggestion. The Introduction has been revised to more explicitly articulate the knowledge gap regarding *Mentha*–rhizosphere interactions. Specifically, we now state that it remains unclear whether closely related *Mentha* taxa grown under similar field conditions harbor distinct rhizosphere microbial communities and how such patterns are associated with soil physicochemical properties and cultivar-specific chemotypes. This clarification has been added immediately prior to the statement of the study aims (Introduction, lines 90–93).

***2. Clearly separate aims (microbiota, soil properties, essential oils, mycorrhiza) from methods.***

Response: We appreciate this helpful suggestion. The statement of aims in the Introduction has been revised to more clearly separate conceptual objectives from methodological approaches. The aims are now presented in a structured format, focusing explicitly on (i) soil physicochemical properties and chemotypes, (ii) rhizosphere microbial community composition and diversity, and (iii) environmental and chemical variables associated with microbial patterns, without reference to specific analytical techniques (Introduction, lines 115–116).

***3. Highlight key contrasts among Mentha taxa more explicitly rather than listing findings.***

Response: The manuscript has been revised to more clearly emphasize the key contrasts among the three *Mentha* taxa. In the Results section, we added a concise synthesis statement in the ASV-level baseline comparison to explicitly note the distinctiveness of *M. spicata* B17 relative to the other cultivars. In addition, the Discussion section now concludes with an integrative summary highlighting that diversity metrics, ordination analyses, and differential abundance results consistently identify *M. spicata* B17 as the most divergent rhizosphere profile, whereas *M. × villosa* B10 and *M. suaveolens* J17 display comparatively more similar microbial patterns (Results: lines 343–345; Discussion: lines 861–863).

***4. Most of figures are not clear, adjust resolution***

Response: The figures have been regenerated at higher resolution and re-uploaded in accordance with the journal’s technical requirements. In particular, Figures 4, 5, 6, and S1 and S2 have been revised and replaced with modified, high-resolution versions to improve clarity and legibility.
