# Supplementary material for: Microbial communities in the rhizosphere of three Mentha species: Links to soil properties and essential oil profiles
Source: PLoS One. 2026 Jul 31;21(7):e0354132. doi: 10.1371/journal.pone.0354132 (PMC13426939; doi:10.1371/journal.pone.0354132)
Supplement: Attachment — Submitted filename: Response_to_Reviewers_auresp_2.docx [file pone.0354132.s010.docx]

**Response to reviewers**:

We thank the Editor and Reviewers for their constructive and insightful comments. We have carefully revised the manuscript accordingly, and all changes are highlighted in the tracked version. Below, we provide a point-by-point response.

**Reviewer 1:**

We thank the reviewer for the careful evaluation of our revised manuscript and for confirming that all previous comments have been adequately addressed. We greatly appreciate the reviewer’s supportive assessment and constructive feedback throughout the review process. No further changes were requested by Reviewer 1.

**Reviewer 2:**

No additional comments from Reviewer 2 were included in the current decision letter; therefore, no further response is required.

**Reviewer 3:**

We thank the reviewer for the additional review and for the comments provided on the revised manuscript. As Reviewer 3 provided comments in annotated form directly within the manuscript PDF, we have addressed them below according to the corresponding manuscript section and comment topic.

***Comment 1 – Abstract: Missing quantitative data, statistical values, diversity indices, functional diversity, and keywords.***

Response: We carefully reconsidered the Abstract in light of this suggestion and checked it against the PLOS ONE guidelines. The Abstract already summarizes the main objectives, the general methodological approach, the most important results, and their significance within the journal’s word limit. Therefore, we did not add detailed statistical values or individual diversity indices, as these are provided in the Results section. Functional diversity was not assessed in this amplicon-based study, and including such information in the Abstract would therefore be misleading. We therefore retained the Abstract in its concise form.

Keywords are provided separately in the manuscript metadata/submission system, as required by the journal (Keywords: *Mentha*; rhizosphere; essential oils; bacterial communities; fungal communities; microbial diversity; medicinal plants).

***Comment 2 – The reviewer suggested improving the technical wording of the aim statement in the Introduction.***

Response: We thank the reviewer for this comment. Although the intended meaning of the original sentence was to describe the comparative objective of the study, we agree that the wording could be improved for clarity and technical precision. We have therefore revised the aim statement in the Introduction. In particular, we replaced the phrase “are associated with differences in the composition and diversity” with clearer wording indicating that we examined whether closely related *Mentha* taxa harbor distinct rhizosphere bacterial and fungal communities. We also replaced “drivers” with “variables statistically associated with” to maintain the correlative framing of the study. See lines 115-121.

***Comment 3 – Single-season sampling. The reviewer asked why the study was based on one sampling season and whether one-season data are reliable.***

Response: We thank the reviewer for raising this point. The aim of the study was not to assess seasonal or year-to-year variation, but to compare rhizosphere bacterial and fungal communities among closely related *Mentha* taxa grown under similar field conditions at the same phenological stage. Therefore, all samples were collected during the full flowering stage in the same growing season to ensure comparability among cultivars. We agree that single-season sampling limits the ability to evaluate temporal stability. Accordingly, we have added a limitation statement to the Discussion, noting that the study represents a season-specific snapshot and that multi-year and multi-stage sampling would be required to assess the temporal stability of the observed patterns. See lines 913–916.

***Comment 4 – References for soil physicochemical analytical methods. The reviewer requested references for the methods used for soil physicochemical analyses.***

Response: The soil physicochemical analyses were performed according to formal Hungarian national standards (MSZ), rather than according to individual research articles. The relevant standard numbers are provided in the Methods section, and the applied standards, together with comparable international standards, are summarized in S1 Table. To make this clearer, we have revised the text to explicitly state that the applied Hungarian national standards and their corresponding or comparable international standards are listed in S1 Table. See lines 150–151.

***Comment 5 – Standard chemicals used for EO compound identification. The reviewer asked which standard chemicals were used for the analysis of essential oils.***

Response: We thank the reviewer for this comment. We have clarified the compound identification procedure in the Methods section. No pure reference standards of individual volatile compounds were used for compound confirmation. Instead, compound identification was based on linear retention indices calculated relative to a homologous series of n-alkanes (C9–C23), mass spectral matching against commercial libraries (NIST and Wiley), and comparison with previously published literature data. No novel compounds were detected, and the identified constituents were consistent with earlier reports on *Mentha* essential oil composition. The corresponding Methods section has been revised accordingly. See lines 178–182.

***Comment 6 – Temperature unit formatting. The reviewer suggested removing the space between numerical values and the temperature unit.***

Response: We checked the formatting of all temperature values throughout the manuscript. As PLOS ONE recommends the use of SI units, temperature values were standardized according to SI unit conventions, using a space between the numerical value and the unit symbol (e.g., 60 °C, 240 °C, 250 °C).

***Comment 7 – 16S rRNA and ITS markers. The reviewer asked whether 16S rRNA sequencing works for fungi.***

Response: We respectfully clarify that 16S rRNA gene sequencing was not used for fungal community analysis. As stated in the Methods section, bacterial community composition was assessed using the 16S rRNA gene, whereas fungal community composition was assessed using the internal transcribed spacer (ITS) region. The following sentences further specify the marker regions and primers used: the V3–V4 region of the bacterial 16S rRNA gene was amplified for bacterial communities, while fungal ITS regions were amplified using ITS1F and ITS4R primers. Therefore, no change was made to this section, as the current wording already distinguishes the bacterial and fungal marker regions. See lines 212–218.

***Comment 8 – Presentation of soil texture / Arany-type plasticity index. The reviewer indicated that the Arany-type plasticity index values should be presented in a table or figure.***

Response: We thank the reviewer for this comment. The Arany-type plasticity index values were reported directly in the Results section because this parameter was used only for soil texture classification. The values showed only a narrow range across the samples (25–27), indicating that all soils belonged to the same sandy loam texture class. Since this parameter was not included in subsequent statistical analyses or correlation analyses, and Table 1 was intended to summarize the main soil physicochemical variables used for group comparisons, we retained the Arany-type plasticity index values in the text rather than adding an additional table row.

***Comment 9 – Table 2 headings. The reviewer commented that the extended table should include headings with other items.***

Response: We thank the reviewer for this comment. We carefully checked Table 2. The table already includes concise column headings, and all abbreviations and units are defined in the table footnote: RT, LRI, and A. LRI are explained, and the footnote states that values represent mean percentage ± standard deviation (n = 3 per cultivar). This format follows common reporting practice in essential oil GC–MS composition tables. We therefore retained the current column-heading structure to preserve readability and avoid overcrowding the table. We also note that the submitted manuscript is not in final typeset layout; therefore, page breaks in the automatically generated review PDF may not reflect the final published formatting. The table will be checked again during production if further formatting adjustments are required.

***Comment 10 – Methodological information in the “Base sequence data information” section. The reviewer asked why methodological information appeared in this Results subsection.***

Response: We thank the reviewer for this comment. We agree that methodological details should be kept in the Materials and Methods section. The introductory sentence in this Results subsection was therefore revised to present the information as a brief description of the sequencing dataset rather than as methodological detail. See lines 317–319. However, we retained the clarification that total ASV counts and Venn analyses were based on the full quality-filtered dataset, whereas diversity indices were calculated after rarefaction, because this distinction was specifically requested during the previous review round and was considered satisfactorily addressed by Reviewer 1. This sentence is necessary to avoid ambiguity in the interpretation of ASV counts and diversity metrics.

***Comment 11 – Formatting of p-values. The reviewer requested changing “P” to “p” throughout the manuscript.***

Response: We thank the reviewer for this comment. The previous notation followed the format that had been requested in one of our earlier PLOS ONE manuscript; however, we agree that the notation should be consistent throughout the present manuscript and aligned with the current statistical reporting style used by PLOS ONE. Therefore, we have checked the manuscript and standardized the notation of p-values throughout the text, tables, and figure captions using lowercase italic p with spaces around comparison operators, e.g., p < 0.05.

***Comment 12 – Cross-references in the text. The reviewer suggested avoiding nonspecific directional references such as “see below” and “see above”.***

Response: We thank the reviewer for this comment. We have checked the manuscript and removed nonspecific directional references such as “see below” where appropriate.

***Comment 13 – Reporting of correlation analyses. The reviewer requested that correlation analyses include r² and p-values.***

Response: We thank the reviewer for this comment. We have revised the reporting of the correlation analyses to provide the corresponding correlation coefficients and significance levels. Because Spearman’s rank correlation was used in this study, the results are reported as Spearman’s correlation coefficients (ρ). In the Results text, the key correlations are now reported together with their corresponding p-values, while in Tables 3 and 4 significance levels are indicated by asterisks. For the RDA analyses, we additionally report R², adjusted R², pseudo-F statistics, and permutation-test p-values for the overall models and explanatory variables where applicable.

***Minor language and formatting comments***

Response: We checked the manuscript for minor language and formatting issues indicated by the reviewer. The sentence referring to AMF inoculation was rephrased for grammatical clarity, and additional minor wording changes were made where appropriate.

We believe that these revisions address the additional comments raised by Reviewer 3 and improve the clarity, consistency, and presentation of the manuscript.
