# Supplementary material for: Microbial communities in the rhizosphere of three Mentha species: Links to soil properties and essential oil profiles
Source: PLoS One. 2026 Jul 31;21(7):e0354132. doi: 10.1371/journal.pone.0354132 (PMC13426939; doi:10.1371/journal.pone.0354132)
Supplement: Peer review history

# Peer review: Decision Letter 0

**DOI:** 10.1371/journal.pone.0354132.r001

**Authors:** Mozaniel de Oliveira

3 Feb 2026

PONE-D-25-58370

Microbial communities in the rhizosphere of three Mentha species: links to soil properties and essential oil profiles

PLOS One

Dear Dr. Juhász,

Thank you for submitting your manuscript to PLOS ONE. After careful consideration, we feel that it has merit but does not fully meet PLOS ONE’s publication criteria as it currently stands. Therefore, we invite you to submit a revised version of the manuscript that addresses the points raised during the review process.

- **I have received the revisions to your manuscript. Please review each point raised by the reviewers.**

-->Please submit your revised manuscript by Mar 20 2026 11:59PM. If you will need more time than this to complete your revisions, please reply to this message or contact the journal office at plosone@plos.org. When you're ready to submit your revision, log on to https://www.editorialmanager.com/pone/ and select the 'Submissions Needing Revision' folder to locate your manuscript file.

Please include the following items when submitting your revised manuscript:

- A letter that responds to each point raised by the academic editor and reviewer(s). You should upload this letter as a separate file labeled 'Response to Reviewers'.
- A marked-up copy of your manuscript that highlights changes made to the original version. You should upload this as a separate file labeled 'Revised Manuscript with Track Changes'.
- An unmarked version of your revised paper without tracked changes. You should upload this as a separate file labeled 'Manuscript'.

If you would like to make changes to your financial disclosure, please include your updated statement in your cover letter. Guidelines for resubmitting your figure files are available below the reviewer comments at the end of this letter.

If applicable, we recommend that you deposit your laboratory protocols in protocols.io to enhance the reproducibility of your results. Protocols.io assigns your protocol its own identifier (DOI) so that it can be cited independently in the future. For instructions see: https://journals.plos.org/plosone/s/submission-guidelines#loc-laboratory-protocols. Additionally, PLOS ONE offers an option for publishing peer-reviewed Lab Protocol articles, which describe protocols hosted on protocols.io. Read more information on sharing protocols at https://plos.org/protocols?utm_medium=editorial-email&utm_source=authorletters&utm_campaign=protocols.

We look forward to receiving your revised manuscript.

Kind regards,

Mozaniel Santana de Oliveira, Ph.D

Academic Editor

PLOS One

**Journal Requirements:**

1. When submitting your revision, we need you to address these additional requirements.

Please ensure that your manuscript meets PLOS ONE's style requirements, including those for file naming. The PLOS ONE style templates can be found at

https://journals.plos.org/plosone/s/file?id=wjVg/PLOSOne_formatting_sample_main_body.pdf and

https://journals.plos.org/plosone/s/file?id=ba62/PLOSOne_formatting_sample_title_authors_affiliations.pdf

2. Thank you for stating in your Funding Statement:

This work was supported by the National Research, Development and Innovation Office of the Hungarian Government (grant no: RRF-2.3.1-21-2022-00007 for Agribiotechnology and precision breeding for food security National Laboratory). Á.J. was supported by the Research Excellence Programme of the Hungarian University of Agriculture and Life Sciences.

Please provide an amended statement that declares *all* the funding or sources of support (whether external or internal to your organization) received during this study, as detailed online in our guide for authors at http://journals.plos.org/plosone/s/submit-now.  Please also include the statement “There was no additional external funding received for this study.” in your updated Funding Statement.

Please include your amended Funding Statement within your cover letter. We will change the online submission form on your behalf.

3. Thank you for stating the following financial disclosure:

This work was supported by the National Research, Development and Innovation Office of the Hungarian Government (grant no: RRF-2.3.1-21-2022-00007 for Agribiotechnology and precision breeding for food security National Laboratory). Á.J. was supported by the Research Excellence Programme of the Hungarian University of Agriculture and Life Sciences.

Please state what role the funders took in the study.  If the funders had no role, please state: "The funders had no role in study design, data collection and analysis, decision to publish, or preparation of the manuscript."

If this statement is not correct you must amend it as needed.

Please include this amended Role of Funder statement in your cover letter; we will change the online submission form on your behalf.

4. Please include your full ethics statement in the ‘Methods’ section of your manuscript file. In your statement, please include the full name of the IRB or ethics committee who approved or waived your study, as well as whether or not you obtained informed written or verbal consent. If consent was waived for your study, please include this information in your statement as well.

5. Please upload a new copy of Figures 4, 5 and S2, as the detail is not clear. Please follow the link for more information:  https://journals.plos.org/plosone/s/figures

6. If the reviewer comments include a recommendation to cite specific previously published works, please review and evaluate these publications to determine whether they are relevant and should be cited. There is no requirement to cite these works unless the editor has indicated otherwise.

[Note: HTML markup is below. Please do not edit.]

Reviewers' comments:

Reviewer's Responses to Questions

**Comments to the Author**

1. Is the manuscript technically sound, and do the data support the conclusions?

The manuscript must describe a technically sound piece of scientific research with data that supports the conclusions. Experiments must have been conducted rigorously, with appropriate controls, replication, and sample sizes. The conclusions must be drawn appropriately based on the data presented.

Reviewer #1: Yes

Reviewer #2: Partly

**********

2. Has the statistical analysis been performed appropriately and rigorously?

Reviewer #1: Yes

Reviewer #2: I Don't Know

**********

3. Have the authors made all data underlying the findings in their manuscript fully available?

The PLOS Data policy requires authors to make all data underlying the findings described in their manuscript fully available without restriction, with rare exception (please refer to the Data Availability Statement in the manuscript PDF file). The data should be provided as part of the manuscript or its supporting information, or deposited to a public repository. For example, in addition to summary statistics, the data points behind means, medians and variance measures should be available. If there are restrictions on publicly sharing data—e.g. participant privacy or use of data from a third party—those must be specified.

Reviewer #1: Yes

Reviewer #2: Yes

**********

4. Is the manuscript presented in an intelligible fashion and written in standard English?

PLOS ONE does not copyedit accepted manuscripts, so the language in submitted articles must be clear, correct, and unambiguous. Any typographical or grammatical errors should be corrected at revision, so please note any specific errors here.

Reviewer #1: Yes

Reviewer #2: No

**********

5. Review Comments to the Author

Please use the space provided to explain your answers to the questions above. You may also include additional comments for the author, including concerns about dual publication, research ethics, or publication ethics. (Please upload your review as an attachment if it exceeds 20,000 characters)

**Reviewer #1:** This manuscript is technically sound and meets PLOS ONE’s publication criteria. The issues raised are limited to clarification and reframing of interpretations rather than additional data collection, and the authors should be able to address them readily. I therefore recommend acceptance after minor revision.

**Reviewer #2:**  the manuscript addresses an interesting and relevant topic, but note that my background does not fully align with the specialized methodologies used.

• I Can provide general scientific and structural feedback, a reviewer with deeper expertise in rhizosphere microbiomics / soil microbial ecology would be better suited for detailed technical assessment.

• My comments are intended to be helpful and constructive within your area of competence.

Explicitly state what is unknown about Mentha–rhizosphere interactions before introducing your study.

Clearly separate aims (microbiota, soil properties, essential oils, mycorrhiza) from methods.

Highlight key contrasts among Mentha taxa more explicitly rather than listing findings.

Most of figures are not clear , adjust resolution

**********

6. PLOS authors have the option to publish the peer review history of their article (what does this mean?). If published, this will include your full peer review and any attached files.

If you choose “no”, your identity will remain anonymous but your review may still be made public.

**Do you want your identity to be public for this peer review?**  For information about this choice, including consent withdrawal, please see our Privacy Policy.

Reviewer #1: No

Reviewer #2: No

**********

[NOTE: If reviewer comments were submitted as an attachment file, they will be attached to this email and accessible via the submission site. Please log into your account, locate the manuscript record, and check for the action link "View Attachments". If this link does not appear, there are no attachment files.]

To ensure your figures meet our technical requirements, please review our figure guidelines: https://journals.plos.org/plosone/s/figures

You may also use PLOS’s free figure tool, NAAS, to help you prepare publication quality figures: https://journals.plos.org/plosone/s/figures#loc-tools-for-figure-preparation.

NAAS will assess whether your figures meet our technical requirements by comparing each figure against our figure specifications.

Attachment Submitted filename: Reviewer Report - PlosOne.pdf

# Author comment: Author response to Decision Letter 1

**DOI:** 10.1371/journal.pone.0354132.r002

6 Feb 2026

Response to reviewers:

We thank the Editor and Reviewers for their constructive and insightful comments. We have carefully revised the manuscript accordingly, and all changes are highlighted in the tracked version. Below, we provide a point-by-point response.

Reviewer 1:

1. Essential oil measurements as proxies for rhizosphere chemistry.

Response: We thank the reviewer for this important and constructive comment. We agree that essential oil (EO) composition determined from aerial plant tissues represents an indirect proxy of rhizosphere chemical conditions and does not directly reflect root exudates, which are the primary mediators at the root-soil interface. In response, we have systematically revised the manuscript to clarify the correlative nature of the EO-microbiome relationships:

Methods section:

We have explicitly stated that EO composition was characterized from aerial tissues and not directly measured in rhizosphere soil or as root exudates. The text now clarifies that associations between EO profiles and microbial communities should be interpreted as correlations with plant chemotype rather than direct measurements of rhizosphere chemical drivers. See lines 183-186

Results section:

The subsection addressing EO-microbiome relationships has been revised to avoid mechanistic phrasing. Expressions implying direct influence (e.g., “drivers,” “shaping effects”) have been replaced with terminology reflecting statistical association (e.g., “associated with,” “aligned with,” “correlated with”).

Discussion section:

Interpretations of EO-related findings have been reframed to emphasize chemotype-associated patterns rather than causal filtering effects. Additionally, a dedicated limitation paragraph has been added at the end of the Discussion explicitly acknowledging that EO measurements represent an indirect proxy and that direct rhizosphere metabolite profiling would be required for mechanistic inference. See lines 901-905

Abstract and Conclusion:

Mechanistic wording has been adjusted to ensure consistent correlative framing throughout the manuscript.

We believe these revisions adequately address the reviewer’s concern and clearly position EO-microbiome relationships as correlative associations with plant chemotype rather than direct evidence of rhizosphere chemical causation.

2. Statistical power and sample size.

Response: We thank the reviewer for highlighting this important point. We acknowledge that the study design (n = 3 biological replicates per cultivar; 12 samples in total) inherently limits statistical power and the precision of effect size estimation.

In response, we have explicitly addressed this limitation in the Discussion by noting that correlations should be interpreted with appropriate caution given the sample size. See lines 906-910. We also clarified that, although multiple analytical approaches (diversity metrics, differential abundance analyses, and multivariate models) yielded consistent trends, expanded spatial or temporal replication would further strengthen generalizability.

3. Multicollinearity in RDA models.

Response: We thank the reviewer for this valuable observation. We acknowledge that variance inflation factors indicated multicollinearity among some predictors retained in the RDA models. In the revised manuscript, we have clarified that the RDA results are interpreted as reflecting combined environmental gradients rather than independent effects of individual variables. Correlated predictors were retained to capture the overall nutrient and chemical context of each cultivar for descriptive purposes. We believe this clarification resolves the concern while preserving the ecological interpretation of the multivariate patterns. See lines 670-672.

4. Definition of bulk soil

Response: We thank the reviewer for this important clarification. We have revised the Methods section to specify the sampling distance from plant rows and to explicitly state that bulk soil samples were collected from mechanically maintained inter-row strips. We now clarify that these samples represent managed, disturbed soil rather than undisturbed natural bulk soil. See lines 142-146

In addition, we have briefly addressed this point in the Discussion, noting that the observed rhizosphere effects should be interpreted as contrasts between plant-associated soil and cultivated inter-row soil. We believe this clarification improves transparency and interpretation of the rhizosphere comparisons. See lines 812-816

Minor comments:

Clarify the rarefaction depth used for sequencing normalization and whether any samples were excluded due to low read counts.

Response: The rarefaction depth used for sequencing normalization has now been explicitly stated in the Methods section. Bacterial 16S datasets were rarefied to 95,742 reads per sample, and fungal ITS datasets to 51,602 reads per sample. No samples were excluded due to low read counts. (See Methods, lines 245–247.)

State the rarefaction depth when reporting ASV counts.

Response: We have clarified this point in the revised manuscript. The total ASV counts and Venn analyses reported in the “Base sequence data information” section are based on the full quality-filtered dataset. In contrast, alpha- and beta-diversity metrics were calculated after rarefaction to 95,742 reads per sample (16S) and 51,602 reads per sample (ITS), as described in the (updated) Methods. This distinction has now been explicitly stated in the Results section for clarity. (See Results, lines 322–325.)

Add sample size (n = 3) to relevant figure captions for transparency.

Response: Sample size information has been added to all relevant main and supplementary figure captions (Figs. 3–8, Figs. S1–S2) for transparency. In addition, sample size (n = 3 per soil type or cultivar) has been specified in the captions of Tables 1 and 2 where mean ± SD values are reported.

For RDA figures, include the percentage of variance explained by each axis and note multicollinearity in the captions.

Response: The percentage of variance explained by RDA1 and RDA2 has been added to the captions of Figures 7 and 8. In addition, multicollinearity among predictors and its interpretation as combined environmental or chemotype-associated gradients are now explicitly noted in the captions. Corresponding numerical values in the Results and Discussion sections have been verified and corrected where necessary to ensure consistency with the RDA plots.

Deposit raw sequencing data in a public repository (e.g., NCBI SRA or ENA) and provide accession numbers.

Response: The raw sequencing data have been deposited in the NCBI Sequence Read Archive (SRA) under BioProject accession number PRJNA1418655. The Data Availability Statement has been updated accordingly.

Reviewer 2:

1. Explicitly state what is unknown about Mentha-rhizosphere interactions before introducing your study.

Response: We appreciate this constructive suggestion. The Introduction has been revised to more explicitly articulate the knowledge gap regarding Mentha–rhizosphere interactions. Specifically, we now state that it remains unclear whether closely related Mentha taxa grown under similar field conditions harbor distinct rhizosphere microbial communities and how such patterns are associated with soil physicochemical properties and cultivar-specific chemotypes. This clarification has been added immediately prior to the statement of the study aims (Introduction, lines 90–93).

2. Clearly separate aims (microbiota, soil properties, essential oils, mycorrhiza) from methods.

Response: We appreciate this helpful suggestion. The statement of aims in the Introduction has been revised to more clearly separate conceptual objectives from methodological approaches. The aims are now presented in a structured format, focusing explicitly on (i) soil physicochemical properties and chemotypes, (ii) rhizosphere microbial community composition and diversity, and (iii) environmental and chemical variables associated with microbial patterns, without reference to specific analytical techniques (Introduction, lines 115–116).

3. Highlight key contrasts among Mentha taxa more explicitly rather than listing findings.

Response: The manuscript has been revised to more clearly emphasize the key contrasts among the three Mentha taxa. In the Results section, we added a concise synthesis statement in the ASV-level baseline comparison to explicitly note the distinctiveness of M. spicata B17 relative to the other cultivars. In addition, the Discussion section now concludes with an integrative summary highlighting that diversity metrics, ordination analyses, and differential abundance results consistently identify M. spicata B17 as the most divergent rhizosphere profile, whereas M. × villosa B10 and M. suaveolens J17 display comparatively more similar microbial patterns (Results: lines 343–345; Discussion: lines 861–863).

4. Most of figures are not clear, adjust resolution

Response: The figures have been regenerated at higher resolution and re-uploaded in accordance with the journal’s technical requirements. In particular, Figures 4, 5, 6, and S1 and S2 have been revised and replaced with modified, high-resolution versions to improve clarity and legibility.

Attachment Submitted filename: Response to Reviewers.docx

# Peer review: Decision Letter 1

**DOI:** 10.1371/journal.pone.0354132.r003

**Authors:** Mozaniel de Oliveira

27 Apr 2026

PONE-D-25-58370R1

Microbial communities in the rhizosphere of three Mentha species: links to soil properties and essential oil profiles

PLOS One

Dear Dr. Juhász,

Thank you for submitting your manuscript to PLOS ONE. After careful consideration, we feel that it has merit but does not fully meet PLOS ONE’s publication criteria as it currently stands. Therefore, we invite you to submit a revised version of the manuscript that addresses the points raised during the review process.

==============================

Dear Dr. Juhász, I have received the reviewers' feedback. Before your manuscript continues processing, you must address the questions raised by one of the reviewers. Review the questions point by point and provide an updated version of your manuscript.

==============================

Please submit your revised manuscript by Jun 11 2026 11:59PM. If you will need more time than this to complete your revisions, please reply to this message or contact the journal office at plosone@plos.org. When you're ready to submit your revision, log on to https://www.editorialmanager.com/pone/ and select the 'Submissions Needing Revision' folder to locate your manuscript file.

Please include the following items when submitting your revised manuscript:

- A letter that responds to each point raised by the academic editor and reviewer(s). You should upload this letter as a separate file labeled 'Response to Reviewers'.
- A marked-up copy of your manuscript that highlights changes made to the original version. You should upload this as a separate file labeled 'Revised Manuscript with Track Changes'.
- An unmarked version of your revised paper without tracked changes. You should upload this as a separate file labeled 'Manuscript'.

If you would like to make changes to your financial disclosure, please include your updated statement in your cover letter. Guidelines for resubmitting your figure files are available below the reviewer comments at the end of this letter.

If applicable, we recommend that you deposit your laboratory protocols in protocols.io to enhance the reproducibility of your results. Protocols.io assigns your protocol its own identifier (DOI) so that it can be cited independently in the future. For instructions see: https://journals.plos.org/plosone/s/submission-guidelines#loc-laboratory-protocols. Additionally, PLOS ONE offers an option for publishing peer-reviewed Lab Protocol articles, which describe protocols hosted on protocols.io. Read more information on sharing protocols at https://plos.org/protocols?utm_medium=editorial-email&utm_source=authorletters&utm_campaign=protocols.

As the corresponding author, your ORCID iD is verified in the submission system and will appear in the published article. PLOS supports the use of ORCID, and we encourage all coauthors to register for an ORCID iD and use it as well. Please encourage your coauthors to verify their ORCID iD within the submission system before final acceptance, as unverified ORCID iDs will not appear in the published article. *Only*  the individual author can complete the verification step; PLOS staff *cannot*  verify ORCID iDs on behalf of authors.

We look forward to receiving your revised manuscript.

Kind regards,

Mozaniel Santana de Oliveira, Ph.D

Academic Editor

PLOS One

**Journal Requirements:**

1. If the reviewer comments include a recommendation to cite specific previously published works, please review and evaluate these publications to determine whether they are relevant and should be cited. There is no requirement to cite these works unless the editor has indicated otherwise.

2. Please review your reference list to ensure that it is complete and correct. If you have cited papers that have been retracted, please include the rationale for doing so in the manuscript text, or remove these references and replace them with relevant current references. Any changes to the reference list should be mentioned in the rebuttal letter that accompanies your revised manuscript. If you need to cite a retracted article, indicate the article’s retracted status in the References list and also include a citation and full reference for the retraction notice.

[Note: HTML markup is below. Please do not edit.]

Reviewers' comments:

Reviewer's Responses to Questions

**Comments to the Author**

1. If the authors have adequately addressed your comments raised in a previous round of review and you feel that this manuscript is now acceptable for publication, you may indicate that here to bypass the “Comments to the Author” section, enter your conflict of interest statement in the “Confidential to Editor” section, and submit your "Accept" recommendation.

Reviewer #1: All comments have been addressed

Reviewer #3: All comments have been addressed

**********

2. Is the manuscript technically sound, and do the data support the conclusions?

The manuscript must describe a technically sound piece of scientific research with data that supports the conclusions. Experiments must have been conducted rigorously, with appropriate controls, replication, and sample sizes. The conclusions must be drawn appropriately based on the data presented.

Reviewer #1: Yes

Reviewer #3: Yes

**********

3. Has the statistical analysis been performed appropriately and rigorously?

Reviewer #1: Yes

Reviewer #3: Yes

**********

4. Have the authors made all data underlying the findings in their manuscript fully available?

The PLOS Data policy requires authors to make all data underlying the findings described in their manuscript fully available without restriction, with rare exception (please refer to the Data Availability Statement in the manuscript PDF file). The data should be provided as part of the manuscript or its supporting information, or deposited to a public repository. For example, in addition to summary statistics, the data points behind means, medians and variance measures should be available. If there are restrictions on publicly sharing data—e.g. participant privacy or use of data from a third party—those must be specified.

Reviewer #1: Yes

Reviewer #3: Yes

**********

5. Is the manuscript presented in an intelligible fashion and written in standard English?

PLOS ONE does not copyedit accepted manuscripts, so the language in submitted articles must be clear, correct, and unambiguous. Any typographical or grammatical errors should be corrected at revision, so please note any specific errors here.

Reviewer #1: Yes

Reviewer #3: No

**********

6. Review Comments to the Author

Please use the space provided to explain your answers to the questions above. You may also include additional comments for the author, including concerns about dual publication, research ethics, or publication ethics. (Please upload your review as an attachment if it exceeds 20,000 characters)

**Reviewer #1:**  I thank the authors for their careful and thorough revision. All major and minor comments from the first review have been addressed with substantive, clearly documented changes. Below I evaluate each point in turn.

MAJOR COMMENTS

1. Essential oil measurements as proxies for rhizosphere chemistry

The authors have systematically revised the manuscript across all relevant sections. The Methods now explicitly state that essential oil composition was characterized from aerial tissues and not directly measured in rhizosphere soil or as root exudates (lines 183–186). Throughout the Results and Discussion, mechanistic language (e.g., "drivers," "shaping effects") has been replaced with correlative terminology (e.g., "associated with," "correlated with"). A dedicated limitation paragraph in the Discussion (lines 901–905) acknowledges this constraint and notes that direct rhizosphere metabolite profiling would be required for mechanistic inference. The Abstract and Conclusion have also been updated for consistent framing. This fully addresses the original concern.

2. Statistical power and sample size

A clear limitation statement has been added to the Discussion (lines 906–910), acknowledging that the replication level inherently limits statistical power and effect size precision. The authors appropriately note that while multiple analytical approaches yielded consistent trends, correlations should be interpreted cautiously, and broader spatial or temporal sampling would strengthen generalizability. This is satisfactory.

3. Multicollinearity in RDA models

The revised manuscript now clearly states that RDA axes are interpreted as representing combined environmental gradients rather than independent effects of individual variables (lines 670–672). The rationale for retaining correlated predictors for descriptive purposes is briefly justified. Figure captions for the RDA plots (Figures 7 and 8) have been updated to include the percentage of variance explained by each axis and to note multicollinearity. This adequately resolves the concern.

4. Definition of bulk soil

The Methods section now specifies the sampling distance (~50 cm from plant rows) and explicitly describes the inter-row strips as managed, disturbed soil maintained by shallow mechanical cultivation (lines 142–146). The Discussion (lines 812–816) frames rhizosphere effects as contrasts between plant-associated soil and cultivated inter-row soil rather than comparisons with pristine background soil. This improves both transparency and the interpretive framework.

MINOR COMMENTS

All minor comments have been addressed satisfactorily:

- Rarefaction depths are now explicitly stated in the Methods (95,742 reads for 16S; 51,602 reads for ITS), with confirmation that no samples were excluded due to low read counts.

- The distinction between total ASV counts (full dataset) and diversity metrics (rarefied dataset) is clearly noted in the Results (lines 322–325).

- Sample sizes (n = 3) have been added to all relevant figure and table captions.

- RDA figure captions now include the percentage of variance explained by each axis and note multicollinearity among predictors.

- Raw sequencing data have been deposited in the NCBI Sequence Read Archive under BioProject accession PRJNA1418655, and the Data Availability Statement has been updated accordingly.

SUMMARY

The authors have been thorough, transparent, and responsive in their revisions. All requested changes have been implemented as genuine reframings and clarifications rather than superficial edits. The correlative language is used consistently throughout the manuscript, statistical limitations are candidly acknowledged, methodological details have been clarified, and the sequencing data are publicly accessible. The experimental design is sound, the methods are clearly described and reproducible, the statistical analyses are appropriate, and the conclusions are well supported by the results. I have no further concerns.

**Reviewer #3:** The topic deals with microbial communities in the rhizosphere of some Mentha species that links to soil

properties and essential oil profiles and worth investigation. However, there are some flaws (see text) that need to be addressed in order to fine-tune the final version of the Ms.

**********

7. PLOS authors have the option to publish the peer review history of their article (what does this mean?). If published, this will include your full peer review and any attached files.

If you choose “no”, your identity will remain anonymous but your review may still be made public.

**Do you want your identity to be public for this peer review?**  For information about this choice, including consent withdrawal, please see our Privacy Policy.

Reviewer #1: **Yes:** Bernard Abakah

Reviewer #3: **Yes:** Diriba Muleta

**********

[NOTE: If reviewer comments were submitted as an attachment file, they will be attached to this email and accessible via the submission site. Please log into your account, locate the manuscript record, and check for the action link "View Attachments". If this link does not appear, there are no attachment files.]

To ensure your figures meet our technical requirements, please review our figure guidelines: https://journals.plos.org/plosone/s/figures

You may also use PLOS’s free figure tool, NAAS, to help you prepare publication quality figures: https://journals.plos.org/plosone/s/figures#loc-tools-for-figure-preparation.

NAAS will assess whether your figures meet our technical requirements by comparing each figure against our figure specifications.

Attachment Submitted filename: PONE-D-25-58370_R1_reviewer 19 March 2026.pdf

# Author comment: Author response to Decision Letter 2

**DOI:** 10.1371/journal.pone.0354132.r004

1 May 2026

Response to reviewers:

We thank the Editor and Reviewers for their constructive and insightful comments. We have carefully revised the manuscript accordingly, and all changes are highlighted in the tracked version. Below, we provide a point-by-point response.

Reviewer 1:

We thank the reviewer for the careful evaluation of our revised manuscript and for confirming that all previous comments have been adequately addressed. We greatly appreciate the reviewer’s supportive assessment and constructive feedback throughout the review process. No further changes were requested by Reviewer 1.

Reviewer 2:

No additional comments from Reviewer 2 were included in the current decision letter; therefore, no further response is required.

Reviewer 3:

We thank the reviewer for the additional review and for the comments provided on the revised manuscript. As Reviewer 3 provided comments in annotated form directly within the manuscript PDF, we have addressed them below according to the corresponding manuscript section and comment topic.

Comment 1 – Abstract: Missing quantitative data, statistical values, diversity indices, functional diversity, and keywords.

Response: We carefully reconsidered the Abstract in light of this suggestion and checked it against the PLOS ONE guidelines. The Abstract already summarizes the main objectives, the general methodological approach, the most important results, and their significance within the journal’s word limit. Therefore, we did not add detailed statistical values or individual diversity indices, as these are provided in the Results section. Functional diversity was not assessed in this amplicon-based study, and including such information in the Abstract would therefore be misleading. We therefore retained the Abstract in its concise form.

Keywords are provided separately in the manuscript metadata/submission system, as required by the journal (Keywords: Mentha; rhizosphere; essential oils; bacterial communities; fungal communities; microbial diversity; medicinal plants).

Comment 2 – The reviewer suggested improving the technical wording of the aim statement in the Introduction.

Response: We thank the reviewer for this comment. Although the intended meaning of the original sentence was to describe the comparative objective of the study, we agree that the wording could be improved for clarity and technical precision. We have therefore revised the aim statement in the Introduction. In particular, we replaced the phrase “are associated with differences in the composition and diversity” with clearer wording indicating that we examined whether closely related Mentha taxa harbor distinct rhizosphere bacterial and fungal communities. We also replaced “drivers” with “variables statistically associated with” to maintain the correlative framing of the study. See lines 115-121.

Comment 3 – Single-season sampling. The reviewer asked why the study was based on one sampling season and whether one-season data are reliable.

Response: We thank the reviewer for raising this point. The aim of the study was not to assess seasonal or year-to-year variation, but to compare rhizosphere bacterial and fungal communities among closely related Mentha taxa grown under similar field conditions at the same phenological stage. Therefore, all samples were collected during the full flowering stage in the same growing season to ensure comparability among cultivars. We agree that single-season sampling limits the ability to evaluate temporal stability. Accordingly, we have added a limitation statement to the Discussion, noting that the study represents a season-specific snapshot and that multi-year and multi-stage sampling would be required to assess the temporal stability of the observed patterns. See lines 913–916.

Comment 4 – References for soil physicochemical analytical methods. The reviewer requested references for the methods used for soil physicochemical analyses.

Response: The soil physicochemical analyses were performed according to formal Hungarian national standards (MSZ), rather than according to individual research articles. The relevant standard numbers are provided in the Methods section, and the applied standards, together with comparable international standards, are summarized in S1 Table. To make this clearer, we have revised the text to explicitly state that the applied Hungarian national standards and their corresponding or comparable international standards are listed in S1 Table. See lines 150–151.

Comment 5 – Standard chemicals used for EO compound identification. The reviewer asked which standard chemicals were used for the analysis of essential oils.

Response: We thank the reviewer for this comment. We have clarified the compound identification procedure in the Methods section. No pure reference standards of individual volatile compounds were used for compound confirmation. Instead, compound identification was based on linear retention indices calculated relative to a homologous series of n-alkanes (C9–C23), mass spectral matching against commercial libraries (NIST and Wiley), and comparison with previously published literature data. No novel compounds were detected, and the identified constituents were consistent with earlier reports on Mentha essential oil composition. The corresponding Methods section has been revised accordingly. See lines 178–182.

Comment 6 – Temperature unit formatting. The reviewer suggested removing the space between numerical values and the temperature unit.

Response: We checked the formatting of all temperature values throughout the manuscript. As PLOS ONE recommends the use of SI units, temperature values were standardized according to SI unit conventions, using a space between the numerical value and the unit symbol (e.g., 60 °C, 240 °C, 250 °C).

Comment 7 – 16S rRNA and ITS markers. The reviewer asked whether 16S rRNA sequencing works for fungi.

Response: We respectfully clarify that 16S rRNA gene sequencing was not used for fungal community analysis. As stated in the Methods section, bacterial community composition was assessed using the 16S rRNA gene, whereas fungal community composition was assessed using the internal transcribed spacer (ITS) region. The following sentences further specify the marker regions and primers used: the V3–V4 region of the bacterial 16S rRNA gene was amplified for bacterial communities, while fungal ITS regions were amplified using ITS1F and ITS4R primers. Therefore, no change was made to this section, as the current wording already distinguishes the bacterial and fungal marker regions. See lines 212–218.

Comment 8 – Presentation of soil texture / Arany-type plasticity index. The reviewer indicated that the Arany-type plasticity index values should be presented in a table or figure.

Response: We thank the reviewer for this comment. The Arany-type plasticity index values were reported directly in the Results section because this parameter was used only for soil texture classification. The values showed only a narrow range across the samples (25–27), indicating that all soils belonged to the same sandy loam texture class. Since this parameter was not included in subsequent statistical analyses or correlation analyses, and Table 1 was intended to summarize the main soil physicochemical variables used for group comparisons, we retained the Arany-type plasticity index values in the text rather than adding an additional table row.

Comment 9 – Table 2 headings. The reviewer commented that the extended table should include headings with other items.

Response: We thank the reviewer for this comment. We carefully checked Table 2. The table already includes concise column headings, and all abbreviations and units are defined in the table footnote: RT, LRI, and A. LRI are explained, and the footnote states that values represent mean percentage ± standard deviation (n = 3 per cultivar). This format follows common reporting practice in essential oil GC–MS composition tables. We therefore retained the current column-heading structure to preserve readability and avoid overcrowding the table. We also note that the submitted manuscript is not in final typeset layout; therefore, page breaks in the automatically generated review PDF may not reflect the final published formatting. The table will be checked again during production if further formatting adjustments are required.

Comment 10 – Methodological information in the “Base sequence data information” section. The reviewer asked why methodological information appeared in this Results subsection.

Response: We thank the reviewer for this comment. We agree that methodological details should be kept in the Materials and Methods section. The introductory sentence in this Results subsection was therefore revised to present the information as a brief description of the sequencing dataset rather than as methodological detail. See lines 317–319. However, we retained the clarification that total ASV counts and Venn analyses were based on the full quality-filtered dataset, whereas diversity indices were calculated after rarefaction, because this distinction was specifically requested during the previous review round and was considered satisfactorily addressed by Reviewer 1. This sentence is necessary to avoid ambiguity in the interpretation of ASV counts and diversity metrics.

Comment 11 – Formatting of p-values. The reviewer requested changing “P” to “p” throughout the manuscript.

Response: We thank the reviewer for this comment. The previous notation followed the format that had been requested in one of our earlier PLOS ONE manuscript; however, we agree that the notation should be consistent throughout the present manuscript and aligned with the current statistical reporting style used by PLOS ONE. Therefore, we have checked the manuscript and standardized the notation of p-values throughout the text, tables, and figure captions using lowercase italic p with spaces around comparison operators, e.g., p < 0.05.

Comment 12 – Cross-references in the text. The reviewer suggested avoiding nonspecific directional references such as “see below” and “see above”.

Response: We thank the reviewer for this comment. We have checked the manuscript and removed nonspecific directional references such as “see below” where appropriate.

Comment 13 – Reporting of correlation analyses. The reviewer requested that correlation analyses include r² and p-values.

Response: We thank the reviewer for this comment. We have revised the reporting of the correlation analyses to provide the corresponding correlation coefficients and significance levels. Because Spearman’s rank correlation was used in this study, the results are reported as Spearman’s correlation coefficients (ρ). In the Results text, the key correlations are now reported together with their corresponding p-values, while in Tables 3 and 4 significance levels are indicated by asterisks. For the RDA analyses, we additionally report R², adjusted R², pseudo-F statistics, and permutation-test p-values for the overall models and explanatory variables where applicable.

Minor language and formatting comments

Response: We checked the manuscript for minor language and formatting issues indicated by the reviewer. The sentence referring to AMF inoculation was rephrased for grammatical clarity, and additional minor wording changes were made where appropriate.

We believe that these revisions address the additional comments raised by Reviewer 3 and improve the clarity, consistency, and presentation of the manuscript.

Attachment Submitted filename: Response_to_Reviewers_auresp_2.docx

# Peer review: Decision Letter 2

**DOI:** 10.1371/journal.pone.0354132.r005

**Authors:** Massimiliano Cardinale

6 Jul 2026

<p>Microbial communities in the rhizosphere of three Mentha species: links to soil properties and essential oil profiles

PONE-D-25-58370R2

Dear Dr. Juhász,

We’re pleased to inform you that your manuscript has been judged scientifically suitable for publication and will be formally accepted for publication once it meets all outstanding technical requirements.

Within one week, you’ll receive an e-mail detailing the required amendments. When these have been addressed, you’ll receive a formal acceptance letter and your manuscript will be scheduled for publication.

An invoice will be generated when your article is formally accepted. Please note, if your institution has a publishing partnership with PLOS and your article meets the relevant criteria, all or part of your publication costs will be covered. Please make sure your user information is up-to-date by logging into Editorial Manager at Editorial Manager® and clicking the ‘Update My Information' link at the top of the page. For questions related to billing, please contact billing support.

If your institution or institutions have a press office, please notify them about your upcoming paper to help maximize its impact. If they’ll be preparing press materials, please inform our press team as soon as possible -- no later than 48 hours after receiving the formal acceptance. Your manuscript will remain under strict press embargo until 2 pm Eastern Time on the date of publication. For more information, please contact onepress@plos.org.

Kind regards,

Massimiliano Cardinale, PhD

Academic Editor

PLOS One

Additional Editor Comments (optional):

Reviewers' comments:

Reviewer's Responses to Questions

**Comments to the Author**

1. If the authors have adequately addressed your comments raised in a previous round of review and you feel that this manuscript is now acceptable for publication, you may indicate that here to bypass the “Comments to the Author” section, enter your conflict of interest statement in the “Confidential to Editor” section, and submit your "Accept" recommendation.

Reviewer #1: All comments have been addressed

**********

2. Is the manuscript technically sound, and do the data support the conclusions?

The manuscript must describe a technically sound piece of scientific research with data that supports the conclusions. Experiments must have been conducted rigorously, with appropriate controls, replication, and sample sizes. The conclusions must be drawn appropriately based on the data presented.

Reviewer #1: Yes

**********

3. Has the statistical analysis been performed appropriately and rigorously?

Reviewer #1: Yes

**********

4. Have the authors made all data underlying the findings in their manuscript fully available?

The PLOS Data policy requires authors to make all data underlying the findings described in their manuscript fully available without restriction, with rare exception (please refer to the Data Availability Statement in the manuscript PDF file). The data should be provided as part of the manuscript or its supporting information, or deposited to a public repository. For example, in addition to summary statistics, the data points behind means, medians and variance measures should be available. If there are restrictions on publicly sharing data—e.g. participant privacy or use of data from a third party—those must be specified.

Reviewer #1: Yes

**********

5. Is the manuscript presented in an intelligible fashion and written in standard English?

PLOS ONE does not copyedit accepted manuscripts, so the language in submitted articles must be clear, correct, and unambiguous. Any typographical or grammatical errors should be corrected at revision, so please note any specific errors here.

Reviewer #1: Yes

**********

6. Review Comments to the Author

Please use the space provided to explain your answers to the questions above. You may also include additional comments for the author, including concerns about dual publication, research ethics, or publication ethics. (Please upload your review as an attachment if it exceeds 20,000 characters)

Reviewer #1: The authors have addressed all my comments. Additionally the material is presented in a very intelligible manner and therefore should be accepted for publication.

**********

7. PLOS authors have the option to publish the peer review history of their article (what does this mean?). If published, this will include your full peer review and any attached files.

If you choose “no”, your identity will remain anonymous but your review may still be made public.

**Do you want your identity to be public for this peer review?**  For information about this choice, including consent withdrawal, please see our Privacy Policy.

Reviewer #1: No

**********

# Editor report: Acceptance letter

**DOI:** 10.1371/journal.pone.0354132.r006

**Authors:** Massimiliano Cardinale

PONE-D-25-58370R2

PLOS One

Dear Dr. Juhász,

I'm pleased to inform you that your manuscript has been deemed suitable for publication in PLOS One. Congratulations! Your manuscript is now being handed over to our production team.

At this stage, our production department will prepare your paper for publication. This includes ensuring the following:

* All references, tables, and figures are properly cited

* All relevant supporting information is included in the manuscript submission,

* There are no issues that prevent the paper from being properly typeset

You will receive further instructions from the production team, including instructions on how to review your proof when it is ready. Please keep in mind that we are working through a large volume of accepted articles, so please give us a few days to review your paper and let you know the next and final steps.

Lastly, if your institution or institutions have a press office, please let them know about your upcoming paper now to help maximize its impact. If they'll be preparing press materials, please inform our press team within the next 48 hours. Your manuscript will remain under strict press embargo until 2 pm Eastern Time on the date of publication. For more information, please contact onepress@plos.org.

You will receive an invoice from PLOS for your publication fee after your manuscript has reached the completed accept phase. If you receive an email requesting payment before acceptance or for any other service, this may be a phishing scheme. Learn how to identify phishing emails and protect your accounts at https://explore.plos.org/phishing.

If we can help with anything else, please email us at customercare@plos.org.

Thank you for submitting your work to PLOS ONE and supporting open access.

Kind regards,

PLOS ONE Editorial Office Staff

on behalf of

Dr. Massimiliano Cardinale

Academic Editor

PLOS One
